# Sub-THz Channel Characterization and Antenna Array Design for 6G Smart Factory Environments at 140 GHz

**DOI:** 10.3390/s26195991

**Published:** 2026-09-22

**Authors:** Cihat Şeker

**Affiliations:** Department of Electrical and Electronics Engineering, Faculty of Engineering and Architecture, İzmir Bakırçay University, 35665 İzmir, Türkiye; cihat.seker@bakircay.edu.tr

**Keywords:** 6G, Sub-THz, 140 GHz, smart factory, ray tracing, Power Delay Profile, antenna array, channel modeling

## Abstract

The ultra-high data rate and ultra-reliable low-latency communication (URLLC) requirements of Industry 4.0 and smart factory applications have made the evaluation of Sub-Terahertz (Sub-THz) frequencies, especially the 140 GHz band, in sixth-generation (6G) wireless networks a considerable research area. Nevertheless, modern literature often makes isotropic assumptions that do not account for the extreme free-space path loss and highly metallic obstacles associated with sub-THz frequencies. To solve this problem, this paper suggests a comprehensive framework combining high-gain directional antenna design with deterministic 3D ray-tracing channel characterization in a realistic industrial environment. Principally, an optimized 140 GHz 4 × 4 high-gain antenna array system was designed and numerically modeled using CST Studio Suite, and its active element pattern (AEP)-based synthesized 3D radiation pattern was then incorporated into a MATLAB-based Shooting and Bouncing Ray (SBR) tool for a more realistic simulation of the antenna system. Procedural generation of a 3D synthetic model of a smart factory, containing metallic obstacles like Computer Numerical Control (CNC) machines, shelving units, and autonomous guided vehicles (AGVs), was conducted to represent the deterministic multipath environment. Path loss, Power Delay Profile (PDP), RMS Delay Spread, and angular dispersion (Angle of Arrival (AoA) and Angle of Departure (AoD)) analysis prove effective for spatial filtering. Despite significant non-line-of-sight (NLoS) obstructions, leading to 106.5 dB of path loss and a high azimuth angle of arrival spread of 82.9°, the design provides a zero outage in the targeted sector of operation. At the same time, directional beamforming tightly controls the azimuth angle of departure spread to 6.5° and RMS delay spread of 21.5 ns. The results demonstrate that using a realistic antenna radiation pattern is critically important. Furthermore, they reveal multipath behavior in complex industrial scenarios and provide vital insights for the deployment of 6G industrial Internet of Things (IIoT) networks.

## 1. Introduction

The exponential development of the Industry 4.0 framework and the exponential growth of Industrial Internet of Things (IIoT) networks have imposed very challenging conditions on future wireless communication systems operating in smart factories. The highly demanding applications like fleet management of Autonomous Guided Vehicles (AGVs), real-time synchronization of ultra-high-resolution digital twins, remote robotics operation, and AR-aided manufacturing require Ultra-Reliable Low-Latency Communication (URLLC) and Tbps-level data rates, which are not attainable by current 5G wireless communication networks [1,2]. In order to satisfy such aggressive requirements, the sixth-generation (6G) wireless networks are shifting towards the untapped opportunities of sub-Terahertz (sub-THz) spectrum compassing between 100 GHz and 300 GHz. Among this frequency range, the 140 GHz band (D-band) is considered as an attractive candidate for 6G IIoT networks due to the availability of a large and continuous bandwidth along with less atmospheric absorption than THz frequencies [3].

However, the use of wireless links operating at 140 GHz in the challenging environment of industrial sites is a great challenge for the physical layer of wireless communication. In the millimeter band of operation, radio waves experience severe Free Space Path Loss (FSPL) and strong absorption and penetration losses [4]. However, even more importantly, the short wavelengths of sub-THz signals make them very sensitive to shadowing by the presence of various obstacles in the path of propagation, including steel columns, metal CNC machines, forklifts, and people working there.

While the highly directional nature of such antennas helps to focus the energy in the transmitted signal on the desired receiver, it changes the characteristics of the multipath components significantly. Waves propagating in rich scattering environments such as smart factories, characterized by a significant amount of metallic clutter, will reflect off storage racks and equipment before reaching the receiver with various delays and angles. While stochastic channel models such as 3GPP TR 38.901 Indoor Factory (InF) are useful generalizations and do give important statistical distributions, they fail to account for the particular deterministic multipath components, such as a strong specular reflection from a particular metallic cabinet, that affect link reliability in the sub-THz band [5]. As a result, 3D Deterministic Ray Tracing (RT) through Shooting and Bouncing Rays (SBR) algorithm has become the gold standard for modeling the sub-THz channels [6].

According to the critical review of the current literature on the topic, there is a significant gap in the RT-based channel modeling for sub-THz bands: most of the existing simulation works assume that all antennas are isotropic and use a simple cosine model for the analytical description of the antenna radiation pattern. This assumption ignores the real properties of the antenna array, such as asymmetry of radiation lobes, side lobe leakages, and different electrical phases. Not taking into account the 3D electromagnetic (EM) radiation pattern results in serious misrepresentations of important channel parameters, including the Power Delay Profile (PDP), Angle of Departure (AoD), and Angle of Arrival (AoA) [7].

The main reason for conducting such a study is due to the strict physical limitations present in the sub-THz regime. In industrial scenarios with many obstacles present in the form of densely packed smart factories, with high free-space path loss and metal obstacles in the scenario, the use of directional antenna arrays becomes a physical necessity. As a result of this, the multipath propagation behavior is heavily dependent on the spatial filtering property of the antenna arrays. The beamwidth, sidelobe leakage, and nulls present in the array are responsible for determining the fate of the scattered rays as to whether they are allowed to propagate or not. With the use of a purely isotropic source assumption at the transmitting antenna, the simulation ignores the spatial filtering property that is inherent in practical directional antennas, hence resulting in many multipath paths that would otherwise have been filtered out in reality. This causes them to predict the time delay spread to be higher than what it actually is. Given such a physical reality, our fundamental concept for research is to incorporate the complete electromagnetic signature of the sub-THz array in a full wave into the digital twin in order to determine the true spatial-temporal channel limits needed for URLLC in 6G.

In order to address this fundamental limitation, this paper offers a comprehensive, deterministic channel modeling framework for 6G smart factories that thoroughly connects antenna design in terms of physics with 3D ray tracing techniques. The key aspects of this research are listed below:

- Designing a 4 × 4 Microstrip Patch Antenna Array for 140 GHz: In designing a 4 × 4 microstrip patch antenna array at 140 GHz, mathematical modeling followed by electromagnetic simulations yields a highly accurate 3D radiation pattern including phase information.
- Industrial Digital Twin Modeling: The digital twin of an intelligent factory is developed in a deterministic manner that includes physical boundaries, steel columns, CNC manufacturing units, metal racks, and moving AGVs.
- Integration of SBR and Physical Antenna Information: The intricate 3D radiation pattern of the designed antenna is incorporated directly within the deterministic SBR ray-tracing module. It provides that multi-bounce rays, spatial filtering, and path loss are all physically controlled by the real directional gain of the transmitting antenna array system, thus removing the inaccuracy of the isotropic transmitter model assumption.
- Detailed Multi-Receiver Channel Characterization: A large number of ray-tracing simulations are performed for 30 randomly chosen positions of the AGVs on the shop floor. Key multipath propagation parameters for sub-THz channels, such as Path Loss vs. Distance Models, Wideband Received Power CDFs, PDP, RMS Delay Spread PDFs, and AoA/AoD angular spreads, are derived and carefully studied.

This study will be organized in the following way. In Section 2, the Related Work will be introduced to point out the theoretical bottlenecks in the field of sub-THz industrial channel modeling. Section 3 will introduce the Technical Background and System Model, which will lay down the mathematical formulation of the digital twin and 140 GHz ray-tracing parameters. In Section 4, the Integrated Electromagnetic Synthesis and Deterministic 3D Ray-Tracing Model will be introduced, highlighting the coupling between the designed antenna and the propagation engine. The spatial-temporal metrics and coverage validations obtained from the analysis will be discussed in Section 5, Results and Discussions. Conclusion will follow in Section 6.

## 2. Related Work

The move to the sub-THz band for industrial use in 6G necessitates a thorough grasp of the processes of channel propagation and antenna behavior. The studies that have been conducted on this topic can be broadly classified into sub-THz channel measurement results, deterministic ray tracing in industrial scenarios, and the influence of realistic antennas on the channel.

### 2.1. Sub-THz Channel Characterization in Industrial Environments

Several measurement campaigns have been carried out in the last couple of years to determine the basic propagation properties of sub-THz waves. Some pioneering works have provided a basis for the models for path loss, scattering, and molecular absorption in W-band and D-band indoors, stressing the significant attenuation effect resulting from atmospheric gases [8,9,10]. In order to extrapolate this empirical knowledge, stochastic models like NYUSIM and 3GPP TR 38.901 Indoor Factory (InF) have been extensively used to model large-scale fading, delay spreads, and angular spreads [11]. Also, recent works employing channel sounders developed for the purpose have been performed to investigate the material reflection and penetration loss at 140 GHz, showing that common materials found in factories like concrete and steel can be nearly perfect reflectors leading to a multipath environment [12]. Other works have been performed in terms of investigating the spatial-temporal multipath clustering phenomenon in confined industrial areas, showing that the sub-THz links are very susceptible to blockages [13]. Nevertheless, although the empirical measurements are very precise, they are site-dependent and labor-intensive. On the contrary, stochastic models offer superb statistical bounds but essentially lack any capability of capturing deterministic multipath effects induced by metal objects, such as CNC machines and storage racks, in an IIoT setting [14].

### 2.2. Deterministic Ray-Tracing for 6G Networks

Deterministic 3D RT, especially the SBR algorithm, has emerged as the go-to solution to counteract the shortcomings of stochastic methods and is now employed to analyze 6G channels site-specifically [15,16,17]. RT has been used in several recent works to study multipath propagation in factories operating at 28 GHz and 60 GHz to emphasize the drastic effect of metal reflections on PDP and delay spread [18]. The modern approaches take advantage of LiDAR point clouds to create highly accurate digital twins required for RT simulations of mmWaves [19,20]. In the sub-THz domain, it has been proven that increasing the frequency results in an exponential drop of multipath components owing to the large penetration losses and diffuse scattering [21]. Also, RT has been effectively applied to deploy RIS in highly blockage-prone sub-THz scenarios [22]. While these results are highly valuable, most RT-based factory channel modeling works still consider only sub-100 GHz bands. The individuals who move into the 140 GHz regime use simplified empty-room models for decreasing the complexity of SBR calculation, thereby ignoring the fact that a very complex multiple reflection situation is created by industrial digital twins [23].

### 2.3. The Antenna Pattern Assumption Gap

The biggest drawback of channel modeling based on RT is the severe oversimplification of the antenna radiation pattern [24,25,26,27]. In order to speed up the computation process of the ray-launching, advanced RT research usually utilizes ideal isotropic radiators or mathematically constructed antenna models (ideal cosine beams or half-wave dipoles). However, at sub-THz frequencies, when highly directional antenna arrays are required to compensate for severe FSPL, such an assumption introduces serious spatial errors. An antenna array at 140 GHz frequency is characterized by distinct side-lobes, back-lobes, array mutual coupling, cross-polarization leakages, and phase characteristics. Recent research on spatial filtering proves that utilization of an accurate 3D electromagnetic pattern in place of a generic antenna theory introduces dramatic changes in the computation of AoA and AoD distribution [28]. The reason lies in the fact that side-lobes of a physical antenna are capable of catching multipath reflections from metallic obstacles, which would be missed by the theoretical cosine beam. Although the phenomenon is well documented, there is no evidence of literature that couples a full-wave simulation of a sub-THz antenna array with a deterministic SBR ray-tracing engine. This paper seeks to address this very crucial methodology deficiency through the incorporation of the 3D complex radiation pattern of the proposed 4 × 4 microstrip patch array into a multireceiver intelligent factory RT simulation.

In the ultra-high-frequency (UHF) used for communication, from coherent optical communication systems to 6G sub-THz technologies, the performance of transceivers is greatly limited by physical layer effects like phase noise and broad Radio Frequency (RF) linewidths. Although recent research has been able to overcome the problem of RF linewidth constraints by using sophisticated machine learning techniques, such as ELM for OFDM [29], handling spatial and temporal dispersion by merely applying digital signal processing consumes a lot of computation. As a compliment to such algorithmic solutions to this problem, the solution we present utilizes the physical filtering properties of a high-gain, highly directional 4 × 4 antenna array to mitigate the effects of multi-bounce at the spatial level, thus preventing multi-bounce delay from occurring prior to the baseband processing stage.

## 3. Technical Background and System Model

This proposed methodology is based on a careful integration of full-wave electromagnetic analysis along with deterministic geometrical optics. This part of the paper presents the formulation of the mathematical framework that describes the environment, physical antenna array, propagation using ray tracing, large-scale fading, and channel characteristics.

### 3.1. 3D Digital Twin and Environment Modeling

To assess the 140 GHz channel propagation properties, an exceptionally accurate 3D digital twin of the Smart Factory is procedurally created in the simulation environment. Denote the Smart Factory space as a Cartesian coordinate system $V\in R^{3}$, with physical space being limited to X∈[−10,10], Y∈[−10,10], and Z∈[0,6] meters.

In contrast to generic empty room-based simulations, the proposed digital twin is filled with concrete industrial clutter, which causes very high multipath scattering. The clutter set C consists of metallic CNC manufacturing cells, numerous steel shelves, a central conveyor belt system, and AGVs (Autonomous Guided Vehicles), which serve as User Equipments (UEs). The boundaries of the environment (floor, walls, ceiling) have the electromagnetic characteristics of typical industrial concrete, while the internal clutter is assumed to be a perfect conductor (metallic material) [30]. It is important to distinguish between the two materials because of the high specularity of sub-THz waves on metallic surfaces and absorption in concrete [31,32].

### 3.2. 140 GHz Antenna Array Formulation

Working at 140 GHz results in an impairment of the link budget caused by the Free Space Path Loss (FSPL) [33]. This is remedied by using a high-gain 4 × 4 planar microstrip patch antenna array for the Transmitter (Tx) Access Point. Considering that the array is mounted in the *xy*-plane, the far-field radiation pattern E(θ,ϕ) is obtained as the sum of the radiation patterns of all the active elements weighted by the Array Factor (AF), which is [34]:

(1) $$E\left(\theta ,\phi \right)=E_{element}\left(\theta ,\phi \right)\cdot \sum_{m=1}^{M}\sum_{n=1}^{N}w_{mn}e^{jk\sin\theta (x_{m}\cos\phi +y_{n}\sin\phi )}$$

here M=4 and N=4 being the number of elements along the *x* and *y* axes, in that order. The complex excitation weight (amplitude and phase) of the *mn*-th element is given by $w_{mn}=a_{mn}e^{j\beta_{mn}}$, k=2π/λ is the wave number at 140 GHz, and $\left(x_{m},y_{n}\right)$ are the physical coordinates of the element centers [35].

Rather than using this idealized mathematical model that, by definition, fails to account for mutual coupling effects, cross-polarization, and surface-wave diffraction [36], we have obtained the actual 3D complex radiation matrix G(θ,ϕ), which includes both amplitude and phase information, by means of a full-wave electromagnetics simulation software. This physical matrix is then interpolated and incorporated directly into the ray tracing model.

### 3.3. 3D Ray-Tracing (SBR) Propagation Framework

At the heart of the propagation analysis lies the Shooting and Bouncing Rays (SBR) technique, an extremely efficient algorithm that combines elements of Uniform Theory of Diffraction (UTD) and Geometrical Optics (GO) [37,38]. The SBR engine launches thousands of discrete ray tubes from the Tx, tracking their interactions with the digital twin environment up to a maximum of $N_{ref}=3$ reflections to balance accuracy and computational complexity.

To optimize the computational efficiency of the 3D ray-tracing engine, a computational ray-pruning threshold ($P_{prune}=-120$ dBm) is applied at the individual ray level. Out of all launched rays, let L denote the exclusive subset of surviving multipath rays whose independent power satisfies $P_{i}^{RX}\geq P_{prune}$. It must be emphasized that this per-ray limit is strictly for computational termination; the actual system-level outage is evaluated later (as detailed in Algorithm 1) by comparing the total coherently summed received power against the absolute hardware receiver sensitivity threshold ($P_{sens}=-90$ dBm). Furthermore, to accurately capture sub-THz propagation dynamics, the model mathematically integrates the molecular absorption phenomenon dictated by atmospheric gases at 140 GHz. Thus, the total received complex electric field $E^{RX}$ at a given AGV location is the coherent summation of all valid rays i∈L, updated as [39]:

(2) $$E^{RX}=\sum_{i\in L}E_{i}^{TX}\cdot \left(\prod_{r=1}^{R_{i}}{\bar{R}}_{i,r}\right)\cdot \left(\prod_{d=1}^{D_{i}}{\bar{D}}_{i,d}\right)\frac{e^{-jkd_{i}}}{d_{i}}e^{-j\Delta \phi_{ant}}e^{-\frac{1}{2}\alpha_{abs}d_{i}}$$

Here, $E_{i}^{TX}$ is the initial electric field of the *i*-th ray launched from the Tx antenna based on the imported full-wave matrix $G(\theta_{D,i},\phi_{D,i})$ [25]. The term $R_{i}$ states the number of reflections for the *i*-th ray, and ${\bar{R}}_{i,r}$ is the dyadic reflection coefficient matrix defined by Fresnel equations for the specific material at 140 GHz [39]. Similarly, ${\bar{D}}_{i,d}$ represents the UTD dyadic diffraction coefficient, $d_{i}$ is the total unfolded path length, and $\alpha_{abs}$ is the power molecular absorption coefficient.

### 3.4. Multipath Channel Metrics and Capacity Calculation

By extracting the deterministic parameters of each ray within the valid subset L (amplitude, phase, delay, and angular trajectory), comprehensive large-scale and small-scale channel metrics are synthesized.

To theoretically frame the severe distance-dependent attenuation and the structural blockage-induced shadowing within the industrial environment, the site-specific Close-in (CI) free space reference distance path loss model is established [8]:

(3) $$PL_{CI}\left(d\right)=PL_{FS}\left(d_{0}\right)+10n\log_{10}\left(\frac{d}{d_{0}}\right)+X_{\sigma }$$

where $PL_{FS}\left(d_{0}\right)$ represents the free space path loss at a reference distance $d_{0}=1$ m, *n* is the Path Loss Exponent (PLE) reflecting the dense metallic clutter of the factory, and $X_{\sigma }∼N\left(0,\sigma^{2}\right)$ is the log-normal shadowing random variable capturing the stochastic Non-Line-of-Sight (NLoS) blockage variations.

The baseband Channel Impulse Response (CIR), h(τ,Ω), characterizing the temporal and spatial multipath behavior, is formulated as [40]:

(4) $$h\left(\tau ,\Omega \right)=\sum_{i\in L}\alpha_{i}e^{-j\Phi_{i}}\delta \left(\tau -\tau_{i}\right)\delta \left(\Omega -\Omega_{A,i}\right)$$

where $\alpha_{i}$, $\Phi_{i}$, and $\tau_{i}=d_{i}/c$ are the amplitude, phase, and time of arrival of the *i*-th multipath component (MPC). $\Omega_{A,i}=\left(\theta_{A,i},\phi_{A,i}\right)$ represents the 3D Angle of Arrival (AoA) [41].

To characterize the temporal dispersion of the channel, which directly impacts Inter-Symbol Interference (ISI) in 6G transceivers, we calculate the Root Mean Square (RMS) Delay Spread ($\tau_{RMS}$) using the discrete Power Delay Profile (PDP), $P\left(\tau_{i}\right)=\alpha_{i}^{2}$, following standard 3GPP definitions [41]:

(5) $$\tau_{RMS}=\sqrt{\frac{\sum_{i\in L}P\left(\tau_{i}\right)\tau_{i}^{2}}{\sum_{i\in L}P\left(\tau_{i}\right)}-{\left(\frac{\sum_{i\in L}P\left(\tau_{i}\right)\tau_{i}}{\sum_{i\in L}P\left(\tau_{i}\right)}\right)}^{2}}$$

In a similar fashion, the spatial diversity and correlation of the multipath signals are also mathematically characterized through the RMS Angular Spread $\left(\sigma_{\theta }\right)$, which characterizes whether or not the use of beamforming arrays is possible. In order to avoid any errors arising out of phase wrapping due to 0°/360° wraparound, the angular parameters have to be carefully calculated based on power-weighted circular statistics. In any arbitrary spatial plane, either Azimuth (*ϕ*) or Elevation (*θ*) plane for either Arrival or Departure, the power-weighted circular mean angle ($\mu_{\theta }$) is first calculated as [39,41]:

(6) $$\mu_{\theta }=\arg\left(\sum_{i=1}^{N}P_{i}e^{j\theta_{i}}\right)$$

where $\theta_{i}$ is the absolute angle (azimuth or elevation) of the *i*-th ray, $P_{i}$ is the received linear power of that ray, and $\mu_{\theta }$ is the power-weighted circular mean angle of the valid multipath rays. This equation resolves the angular discontinuities in the directional quantities by expressing them in terms of their equivalent values on the complex plane using $e^{j\theta_{i}}$.

Finally, the ultimate metric for 6G industrial deployments is the achievable channel capacity. Assuming an Additive White Gaussian Noise (AWGN) floor dominated by thermal noise $N_{floor}=k_{B}TBF$, the theoretical Shannon-Hartley channel capacity *C* (in bits per second) for a given location is computed as [42]:

(7) $$C=B\log_{2}\left(1+\frac{P_{RX}}{N_{floor}}\right)$$

where *B* is the allocated sub-THz bandwidth, *F* is the receiver noise figure, and $P_{RX}=\sum_{i\in L}P\left(\tau_{i}\right)$ is the total wideband received power obtained from the deterministic SBR simulation. The total noise floor $N_{floor}$ is explicitly defined as $-174\;\mathrm{dBm}/\mathrm{Hz}+10\log_{10}B+NF$. Assuming the system bandwidth is 2 GHz and the noise figure of the receiver is 10 dB, the thermal noise floor for our simulations is determined to be −71 dBm.

Table 1 provides an elegant way of summarizing the cause-and-effect relations behind the deterministic propagation model. Since each mathematical expression has been matched with the corresponding physical phenomenon in the smart factory, the proposed theoretical model removes the ambiguity that stochastic models suffer from. In particular, full-wave spatial filtering together with sub-THz molecular absorption serves as a natural physical constraint that filters out only a limited number of multipaths. The deterministic reduction in metallic higher-order reflections is the reason why overestimation of temporal dispersion by isotropic models cannot occur. Therefore, the created metrics accurately convert the stringent physics of signal propagation into measurable limits for assessing the physical-layer feasibility of 6G URLLC systems.

## 4. Integrated Electromagnetic Synthesis and Deterministic 3D Ray-Tracing Model

For the purpose of validating the proposed model, a complete simulation approach was formulated in this research work. Firstly, a microstrip patch antenna operating at 140 GHz was designed and simulated in the electromagnetic simulation environment. Afterward, the simulation data was transferred to the channel modeling environment, and the channel parameters were derived. This chapter is devoted to describing the electromagnetic simulation environment, the channel modeling environment using the ray-tracing technique, environmental parameters, and simulation steps.

### 4.1. Overview

The methodology adopted by this study successfully incorporates electromagnetic hardware synthesis and deterministic propagation models within a robust cause-and-effect analysis in order to combat the extremely high free space path loss present in the 140 GHz sub-THz band. As clearly demonstrated in the initial step of the overall flowchart illustrated in Figure 1, the customized design of a highly directive 4 × 4 microstrip patch antenna array within CST Studio Suite was used to effectively counter the unrealistic isotropic antenna design that exists extensively in the literature. In order to avoid unnecessary computational burden during the simulation, the Active Element Pattern (AEP) synthesis approach was applied, resulting in the successful extraction of a practical 3D radiation pattern with a maximum gain of 16.86 dBi. Based on the high spatial filtering capabilities of the particular antenna designed, any physically impossible multi-bounce reflection cannot be included in the ray-tracing model, and thus providing a rigorous foundation for accurate channel characterization. Thus, the robustness of the hardware against any kind of metallic scattering that might occur in the environment is assured from the beginning.

The second and third stages of Figure 1 present the smart factory digital twin and the deterministic propagation engine, respectively, which were specially designed to accurately represent real-world scenarios. The complex industrial scenario, under which sub-THz wave propagation experiences strong scattering, was simulated with MATLAB R2024a in a 20 × 20 × 6 m^3^ scenario with a high concentration of metallic CNC machines, storage racks, and Autonomous Guided Vehicles (AGVs). Given that sub-Terahertz waves rapidly attenuate after each reflection, their energy is below the thermal noise floor after three reflections. Given such a physical limitation, the upper bound reflection value of the SBR algorithm was constrained to three, thus significantly decreasing computational costs without decreasing physical fidelity. In contrast with stochastic models, this 3D simulation engine allows for an analytical compensation for 140 GHz free-space path losses and material absorption, allowing for geometry-based and completely deterministic extraction of the geometrical path. Therefore, it becomes possible to analyze in depth the effects of huge numbers of reflections and diffraction at the manufacturing facility.

In the fourth and last phase of Figure 1, a detailed characterization of the channel in both space and time domains is performed using the data gathered from the integrated simulation engine. The huge difference in the physical properties of the extremely narrow Angle of Departure (AoD), as limited by the high directivity of the antenna, and that of the wide AoA due to the surrounding metallic machinery is shown quantitatively via angular spread analysis. Although there is metallic obstruction present, the directional nature of beamforming eliminates unnecessary reflected paths, thereby keeping the root-mean-square (RMS) delay spread confined within a very narrow range. This time constraint becomes the most important physical phenomenon in addressing the requirement of low latency in 6G networks. In all, by providing precise mapping of high-frequency large-scale fading losses and coverage dynamics, this deterministic approach confirms the capability of the sub-THz band for assessing the 6G URLLC physical-layer with high scientific rigor in modern scientific literature.

### 4.2. Full-Wave Antenna Array Design

The antenna was designed as an inset fed microstrip patch antenna with physical dimensions of 0.62mm×0.72mm. The entire design was simulated using the time domain solver in CST Studio Suite. Rogers 3003 material was selected as the substrate with a relative permittivity ($\varepsilon_{r}$) of 3.0 and loss tangent (tanδ) of 0.001. Copper was employed as the conductive layer, featuring a conductivity of $5.8\times 10^{7}\;S/m$ and a thickness of $1.7\times 10^{-5}\;m$. To evaluate its performance, the antenna was simulated at a center frequency of 140 GHz using CST’s Finite Integration Technique (FIT)-based solver. The suggested antenna’s reflection coefficient ($S_{11}$), −10dB bandwidth, radiation pattern, and gain were meticulously plotted. The antenna design, including dimensions and inset-feed configuration, is presented in Figure 2, and the dimensions and parameters of the proposed antenna are resumed in Table 2. Material properties and measurement parameters were precisely selected. Thus, the aim was to achieve optimum performance in an indoor factory environment in the 140 GHz frequency band.

In highly dense 6G sub-THz communication scenarios, simulating a complete large-scale antenna array with its entire feeding network using CST Studio Suite is computationally prohibitive and practically unnecessary for channel modeling purposes. To accurately reflect the radiation characteristics while avoiding massive computational overhead, this study employs the Active Element Pattern (AEP) synthesis method. In this approach, the optimized 140 GHz single patch antenna is simulated in a finite array environment to capture mutual coupling and edge effects. The full 4 × 4 array radiation pattern is then analytically synthesized by multiplying this active element pattern with the corresponding Array Factor (AF), assuming a uniform corporate feed network. The geometry of the array is characterized by a constant spacing of d=λ/2 (1.07 mm at 140 GHz). In order to obtain maximum broadside directivity, it is assumed that there is a uniform distribution of amplitudes with a phase of 0 degrees for all sixteen ports in the full-wave simulation. The single active element microstrip antenna demonstrates a high efficiency of 81.5% of radiation in the full-wave simulations. This method guarantees a highly realistic 16.86 dBi peak gain and 3D directivity profile, which is directly exported to the MATLAB ray-tracing algorithm to evaluate realistic path loss and channel capacity.

### 4.3. Digital Twin Construction

In the present research, an extremely cluttered virtual smart factory environment having a size of 20 m by 20 m by 6 m was designed on the MATLAB simulation software. The industrial digital twin is characterized by a set of macroscopic scatterers such as Computer Numerical Control (CNC) machines, metallic shelving units, and a conveyer belt, using particular material characteristics such as perfectly conducting metal and concrete, together with a 140 GHz access point and 30 Autonomous Guided Vehicles (AGVs) as user equipment, which is described in Figure 3. The floor topology of the factory is flat, with metallic objects that are dense in nature, compared to geographical elevation changes. The spatial capacity heatmap illustrated in Figure 3 was not interpolated using just 30 data points; instead, it has been mathematically calculated on a fine-grained spatial grid covering the whole factory floor area. The 30 separate AGVs were deliberately deployed as User Equipments (UEs) to obtain the required Angle of Arrival/Departure (AoA/AoD) and delay spread measurements in the target region, and not for determining the global coverage map. The particular case study helped in carrying out the deterministic SBR algorithm precisely. Consequently, the propagation characteristics of the 140 GHz sub-THz band, specifically spatial coverage and multipath dispersion, were investigated to validate its feasibility for physical-layer of 6G Ultra-Reliable Low-Latency Communication (URLLC) networks.

Figure 3 indicates the 3D digital twin of the smart factory environment and the ground distribution of the 140 GHz channel capacity. The spatial arrangement of all industrial clutter and autonomous vehicles on the factory floor was deterministically constructed in MATLAB to reflect a realistic manufacturing layout. The target autonomous guided vehicle (AGV) acting as the receiver (Rx) is shown as a green box, and the idle AGVs are shown as yellow boxes, while the fixed 140 GHz access point (Tx Array) is represented by a red downward-pointing triangle. The ground is visualized as a spatial heatmap representing the calculated channel capacity in Gbps. The macroscopic scatterers are distinctly color-coded: the storage racks are orange, the CNC production cells are translucent light gray, the conveyor system is dark gray, and the structural pillars are solid gray. The highly directional radiation pattern emitted by the transmitter is illustrated as a translucent yellow cone. Moreover, the straight LoS propagation channel between the access point and target AGV is designated by a solid red line. The unlit dark zones on the heat map are not unplanned outages; they are the deliberate physical outcome of the very directional nature of the 4 × 4 array. The spatial variation on the floor heatmap plane is directly dependent on the strength of the received signal and the Shannon-Hartley Capacity Model.

In the model development, the AGVs operating as the user equipment are placed at a height of 1.0 m, depending on the 6G Industrial Internet of Things (IIoT) system’s specifications. The height is indicative of the antenna installation height for an industrial mobile robot. The AGV receiver can be assumed to behave like an isotropic antenna (0 dBi) so as to serve as an unbiased spatial probe. Making sure all the multipath signals are received without spatial filtering on the receiver end. The carrier frequency for 6G sub-THz systems is considered in the simulation process with a focus on the D band with a center frequency of 140 GHz. Given that signals within the sub-THz region face high free space path loss and molecular attenuation, directional 4 × 4 microstrip patch antennas are highly recommended over the ideal omnidirectional antennas to provide precise spatial filtering. From a computational point of view, the highly divergent multipath components are pruned preemptively based on a sensitivity threshold of −120 dBm. Next, from the perspective of the receiver, the outage of the entire system is critically assessed using the stringent threshold of −90 dBm sensitivity, which only applies to the sum of received power. Those below the threshold will be drowned out by the thermal noise floor. Therefore, the channel is described based on real-life boundary conditions. To describe the output power of the transmitter for a realistic high-frequency indoor factory access point, the value of 10 dBm is defined. The power value, together with the high-gain directional beam, provides an opportunity to consider all the severe path loss and multiple reflection effects that may occur in a metallic industrial environment. Additionally, it helps to optimize Shooting and Bouncing Rays (SBR) algorithm by limiting the number of reflections and achieving balance between accuracy and coverage mapping. All the aforementioned technical parameters allow modeling the system structure in accordance with physical-layer of 6G URLLC necessities and the cluttered factory environment.

### 4.4. Three-Dimensional Ray-Tracing Simulation Execution

In this study, a channel model based on the technique of ray tracing is used for modeling the 6G IIoT channel parameters in an environment of a highly cluttered smart factory. In this context, the deterministic technique of shooting and bouncing rays (SBR) is adopted. Under the SBR technique, the interaction of electromagnetic waves with the surfaces of the environment is computed using the Geometrical Optics (GO) technique. The rays of the signals are considered to emanate from the highly directional transmitter antenna array. It is considered that the emanated rays from the transmitter strike and bounce off from the surroundings, especially metal objects. All the possible routes by which the signal reaches the receiver at the target AGV are determined. The highest number of reflections possible in the model is considered to be three. This figure is very important when considering the sustainability of communications in the face of difficult non-line-of-sight (NLoS) conditions caused by the use of heavy machinery. This is the case since in NLoS conditions, the signal reaches the receiver via reflection only. The maximum reflection limit of three ensures high-fidelity deterministic simulation model since, on one hand, the most important multipath effects are considered, while on the other hand, the amount of processing required remains manageable since sub-THz signals always lie below the thermal noise floor after three reflections due to extremely high path loss. Therefore, the path loss effect, specular reflection, and dependence on the spatial direction, which are peculiar for the 140 GHz 6G signal propagation, are carefully simulated. The analysis was performed in a 3D procedural digital twin of a smart factory of dimensions $20\times 20\times 6\;m^{3}$, which was created in the MATLAB environment and includes many metallic obstacles. In order to evaluate the high-fidelity deterministic simulation model of the sub-THz band, specific macroscopic scatterers such as CNC machines and storage racks with ITU-R compliant material properties were contained in the system.

The deterministic ray-tracing approach considers the influence of industrial environment structures on the signal direction and power. For this reason, the estimation of the coverage area becomes much more accurate in comparison with traditional stochastic models. The main benefit of the deterministic ray-tracing algorithm is its ability to conduct location-based channel estimation by taking into consideration the geometry of the smart factory. It is an essential condition for short-range, highly directional communication and high-frequency sub-THz in 6G IIoT networks. Precise coverage analysis and realistic channel modeling of the highly cluttered indoor factory make this research more accurate and practical. Figure 4 presents the 3D ray-tracing trajectories generated by the deterministic SBR algorithm between the 140 GHz access point and a sample AGV location. Parameters used in the deterministic SBR-based channel modeling simulation conducted using MATLAB software are tabulated in Table 3. Likewise, the complex permittivity and conductivity of the materials in the digital twin of an industrial scenario in MATLAB software are derived from the ITU-R P.2040-4 technical report for the Indoor Factory (InF) scenario and are tabulated in Table 4.

In order to mathematically justify the computationally optimal bounds of the SBR engine and remove any model-based assumptions, a thorough ray-tracing convergence analysis was performed using maximum reflection limit values of $N_{max}=1$ to 5. The results showed that whereas the first- and second-order reflections represent the major signal energy, third-order bounces were absolutely essential in ensuring multipath profile stability in clutter-rich regions, resulting in the key values of the Deep NLoS metrics, which are equal to −64.7 dBm for power and 21.5 ns for RMS delay spread. Most importantly, an additional convergence analysis based on four and five reflections demonstrated that an energetic and temporal convergence plateau has been achieved beyond which no significant change occurred. More specifically, for N≥4, there was no change in the total received power higher than 0.1 dB, and the RMS delay spread change did not exceed 0.1 ns. This fast stabilization is a straightforward result of the physics behind the 140 GHz transmission phenomenon: rays having four or more reflections follow relatively large unwrapped geometric path lengths, hence being exposed to enormous Free Space Path Loss (FSPL). When this already enormous FSPL is combined with the additional losses resulting from absorption and reflection at each consecutive impact, the power level of these high-order rays definitely drops below the effective noise floor level of −71 dBm and the receiver sensitivity level of −90 dBm. Therefore, the three-reflection $N_{max}=3$ truncation is applied not as an empirical hypothesis but as a physically justified boundary condition that allows one to maintain determinacy of the spatiotemporal channel model while avoiding the exponential complexity of the higher-order simulations.

### 4.5. 140 GHz Received Signal Power Modeling

In this work, an extensive investigation of various parameters that affect the received signal strength was conducted in the context of the smart factory with heavy cluttering. The above factors include excessive free space path loss (FSPL), intense multipath phenomena caused by the presence of reflective metallic objects, and the frequency-dependent molecular absorption nature of the sub-THz communication channel. In this way, a better deterministic model for the reception of signals in 6G Industrial IoT was obtained through the analysis of the above factors, considering the spatial filtering capabilities of the customized 4 × 4 microstrip patch antenna array.

#### 4.5.1. Path Loss

Path loss can be defined as the loss suffered by the electromagnetic waves while traveling from a 140 GHz access point that is fixed in position to the 30 AGVs. The THz frequency sub-band plays a crucial role in facilitating Terabit per second data rates for 6G Industrial Internet of Things (IIoT), but it is very vulnerable to environmental scattering and molecular absorptions. Thus, path loss becomes an important factor. In the smart factory setting, where there is dense metallic clutter with CNC machines, shelves, and concrete buildings, the signal suffers from strong reflections, diffractions, and shadowing. In such a case, the path loss is expected to be extremely variable both in the case of LoS as well as NLoS scenarios. This study applies a deterministic SBR ray tracing approach in which consideration is given to the distance traveled by the signal in each AGV scenario, the material properties of the surfaces as per ITU-R P.2040, and environmental blockages. A path loss model based on a deterministic approach is developed for the specific site in this context. As stated in the 3GPP TR 38.901 technical report for the Indoor Factory (InF) scenario, the close-in (CI) path loss model has been derived using:

(8) $$PL_{CI}\left(d\right)=PL_{FS}\left(d_{0}\right)+10n\log_{10}\left(\frac{d}{d_{0}}\right)+X_{\sigma }$$

here $PL_{CI}\left(d\right)=$ total path loss (dB) at 3D distance *d* in meters between the transmitting array and the AGV receiver, whereas $PL_{FS}\left(d_{0}\right)$ is the free space path loss corresponding to the reference distance $d_{0}$, which is typically 1 m and is given by the following expression according to the Friis transmission equation:

(9) $$PL_{FS}\left(d_{0}\right)=20\log_{10}\left(f\right)+20\log_{10}\left(d_{0}\right)-147.55$$

where *f* denotes frequency in Hertz ($140\times 10^{9}$ Hz). The constant 147.55 is derived from the speed of light and unit conversions. *n* denotes Path Loss Exponent (PLE), which depends on the physical properties of the industrial environment of propagation. Although it is in general about 2 in the open free space environment, it varies significantly in the Indoor Factory environment, based on the degree of clutter (e.g., InF-SL and InF-DL). $X_{\sigma }$ is the zero-mean normally distributed random variable, which corresponds to the severe shadowing impacts occurring randomly because of many NLoS blocks. Generally, its standard deviation *σ* is uniquely specified for metallic industrial terrains. It is known that the strength of the received signal not only depends on the distance but also on the geometrical and physical properties of the metallic reflecting medium. This deterministic approach ensures much more precision than the classical stochastic approach based on the log-distance formulation.

#### 4.5.2. Received Power

The received signal power ($P_{r}$) is estimated based on several factors, including 10 dBm transmit power of the transmitter, 3D gains of the bespoke 4 × 4 microstrip patch antenna array, high sub-THz path loss, effects of the environment (mostly metallic specular reflections and scattering), attenuation based on the reflection limits, and the spatial position of the AGV receiver. Within this context, the total received signal power can be considered as the sum of vectors of the multipaths as:

(10) $$P_{r}={\left|\sum_{i=1}^{N}\sqrt{P_{t_{i}}}\cdot G_{t_{i}}\cdot G_{r_{i}}\cdot e^{-j\phi_{i}}\cdot \frac{\lambda }{4\pi d_{i}}\cdot L_{i}\right|}^{2}$$

where, $P_{r}=$ total received power at the AGV terminal; $P_{t_{i}}=$ transmit power of the *i*th path from the transmitter; $G_{t_{i}}$ and $G_{r_{i}}$ = antenna gain of the highly directional transmitter and receiver in the *i*th path direction in that order (where spatial filtering causes attenuation of off axis waves); $\phi_{i}=$ phase shift of the *i*th path; *λ* = wavelength of the carrier (≈2.14 mm); $d_{i}=$ path distance of the *i*th path; $L_{i}=$ total attenuation coefficient of the *i*th path consisting of specular reflection loss and molecular absorption loss in sub-thz region; *N* = number of surviving paths found by the SBR ray tracing algorithm; and *j* = imaginary number. In this work, the outage condition is clearly defined by the threshold of absolute receiver sensitivity of −90 dBm. Effective data rates are determined by the continuous Shannon capacity equation only for areas with $P_{RX}\geq -90$ dBm. However, any point on the geographical map that has a received power level lower than this threshold ($P_{RX}<-90$ dBm) is unambiguously considered as experiencing an outage condition. Despite the mathematical existence of the capacity according to the theoretical Shannon-Hartley channel, the actual practical capacity at this signal level is considered as 0 bps/Hz. The physical basis for defining the outage condition in such a manner is that a signal level so much lower than the −71 dBm thermal noise level precludes any successful basic modem synchronization process. In addition, it should be noted that the conversion from the physical signal accessibility level to the actual URLLC reliability performance (e.g., BLER or MCS throughput) needs a complete link-level evaluation, which is beyond the scope of the channel model in this manuscript. In the MATLAB platform, the coverage quality for favorable, low-dispersion propagation foundation URLLC was assessed through determination of the power received in the model at each of the 30 locations of AGVs in the smart factory.

To conduct an exact reproduction in the Shannon capacity and received power evaluation, all the system parameters are defined. The bandwidth is chosen as B=2 GHz, while the environmental temperature is T=290 K, and the noise figure of the receiver equals NF=10 dB, thus offering a thermal noise power of −71 dBm. Unlike the use of the simplistic Friis equation for received power ($P_{RX}$) in the considered 3D ray-tracing model, it is determined by coherent addition of all paths. The radiation pattern of each *i*-th ray is individually weighted by the complex AEP-based synthesized 3D pattern 4 × 4 transmitter antenna array (16.86 dBi peak gain at 10 dBm transmitted power). On the other hand, for defining the physical nature of the environment without any hardware bias, the Path Loss values provided in this work indicate the spatial channel loss in an omnidirectional manner. The channel path loss includes the FSPL as well as the exact absorption and reflection coefficient of the materials used, excluding any spatial directivity of the antennas so that there will be no double counting.

#### 4.5.3. Delay Spread Analysis

The exact path length of each multipath element takes to reach each of the 30 AGVs from the 140 GHz access point that is at a fixed location in the simulations was obtained using the deterministic ray tracing technique. Propagation delays were then obtained based on the propagation velocity of the wave. This is done using the formula below:

(11) $$\tau_{i}=\frac{d_{i}}{c}$$

where $\tau_{i}$ is the total time delay of the $i^{\mathrm{th}}$ signal propagation path (seconds), $d_{i}$ is the distance of the path (meters), and *c* is the speed of propagation of electromagnetic waves in a vacuum ($3\times 10^{8}$ m/s). It was observed in the time domain that the dominant LoS components have the least delays and carry the most energy at the receiver. Nevertheless, it is important to note that in the 140 GHz frequency band, the severe attenuation due to reflection naturally restricts the existence of high-order multipaths. This results in a very narrow RMS delay spread, which satisfies the 6G URLLC latency requirements.

#### 4.5.4. Angular Spread Analysis

In addition to the dispersion in the time domain, the spatial diversity of multipath components over the 30 different AGV positions is basically controlled by the angular spread of the Angle of Departure (AoD) and Angle of Arrival (AoA). For a strict evaluation of spatial 3D correlation, and therefore ensuring the feasibility of the proposed beamforming schemes in 6G communications, it becomes extremely important to separate spatial planes for calculating angular spread in Azimuth and Elevation planes. Moreover, since angles are directional quantities, using standard Euclidean calculations results in inaccurate evaluations close to the 0°/360° point. Thus, to overcome phase wrapping issues, the RMS angular spread values are calculated using well-defined circular statistics, and are given by:

(12) $$\mu_{\theta }=\arg\left(\sum_{i=1}^{N}P_{i}e^{j\theta_{i}}\right)$$

(13) $$\sigma_{\theta }=\sqrt{\frac{\sum_{i=1}^{N}P_{i}{\left[\Delta (\theta_{i},\mu_{\theta })\right]}^{2}}{\sum_{i=1}^{N}P_{i}}}$$

(14) $$\Delta \left(\theta_{i},\mu_{\theta }\right)=\mathrm{mod}\left(\theta_{i}-\mu_{\theta }+\pi ,2\pi \right)-\pi$$

where $\theta_{i}$ is the absolute value of the arrival or departure angle of the *i*-th multipath component, $P_{i}$ is the respective linear received power, and *N* is the number of all surviving multipath components. The power-weighted circular mean angle $\mu_{\theta }$ is estimated in the complex plane (Equation (13)) and is naturally free from the angular boundary problem due to computation in the complex plane. After that, the RMS angular spread ($\sigma_{\theta }$) is rigorously calculated (Equation (14)), with the help of the modulo operation, $\Delta (\theta_{i},\mu_{\theta })$ shown in Equation (15), ensuring the extraction of the shortest angular difference between the two values through wrapping all angles into the [−π,π] range. Performing such a calculation procedure separately, one can get four different spatial measurements: Azimuth Spread of Arrival (ASA), Azimuth Spread of Departure (ASD), Elevation Spread of Arrival (ESA), and Elevation Spread of Departure (ESD).

This is detailed in Algorithm 1, which describes the developed deterministic model, integrating the synthesis of high-precision electromagnetic hardware with sub-THz propagation physics. Realizing that dependence on only isotropic antennas leads to an overestimation of spatial spread, the 3D AEP-synthesized radiation pattern of the directional 4 × 4 antenna array is incorporated into the model to ensure spatial filtering. Following this, the deterministic SBR algorithm computes the reflection geometry of several reflections for 30 different AGV locations in the digital twin. After the first stage of pruning the diverged rays using computations (rays that are significantly diverged from the desired values are pruned based on the value of $P_{prune}$), all multipath components up to the reflection limit of $N_{max}=3$ are included to create the full Power Delay Profile (PDP). Importantly, the sensitivity level of the receiver of −90 dBm used at the system level is not applied to individual rays; instead, it is calculated only in the last step of computations when the total wideband power is coherently summed, and the outage is determined. Hence, the sparsity in time-domain metrics is essentially a physical phenomenon due to severe 140 GHz attenuation in free space and starvation in space caused by the directional transmission.
**Algorithm 1** Deterministic 140 GHz Spatial-Temporal Channel Characterization Framework**Require:** Carrier Frequency f=140 GHz, Tx Power $P_{t}=10$ dBm, Rx Sensitivity $P_{sens}=-90$ dBm, Ray Pruning Threshold $P_{prune}=-120$ dBm, Max Reflections $N_{refl}=3$**Require:** Smart Factory Digital Twin Data (CNCs, Racks, Concrete, Metals) with ITU-R P.2040 material properties**Ensure:** Path Loss ($PL_{CI}$), Received Power ($P_{r}$), RMS Delay Spread ($\tau_{RMS}$), Angular Spreads ($\sigma_{AoA},\sigma_{AoD}$), System Outage      *// Phase 1: Full-Wave Electromagnetic Synthesis*  1:$Antenna_{Patch}\leftarrow \mathrm{SIMULATE}\_\mathrm{IN}\_\mathrm{CST}\left(\mathrm{Rogers}\;\mathrm{RO}3003,140\;\mathrm{GHz}\right)$  2:$Pattern_{3D}\left(\theta ,\phi \right)\leftarrow \mathrm{EXTRACT}\_\mathrm{AEP}\_\mathrm{SYNTHESIS}\left(Antenna_{Patch},4\times 4\;\mathrm{Grid}\right)$                       ▹ Yields 16.86 dBi peak gain      *// Phase 2: Digital Twin Initialization*  3:$E_{Twin}\leftarrow \mathrm{INITIALIZE}\_\mathrm{MATLAB}\_\mathrm{ENVIRONMENT}\left(20\times 20\times 6\;m^{3}\right)$  4:$\mathrm{DEPLOY}\_\mathrm{TRANSMITTER}(Loc_{Tx},\mathrm{Height}=5.5\;m,Pattern_{3D}\left(\theta ,\phi \right))$  5:$U_{AGV}\leftarrow \mathrm{DEPLOY}\_\mathrm{RECEIVERS}\left(N=30,\mathrm{Height}=1.0\;m\right)$      *// Phase 3 & 4: Deterministic SBR Engine & Channel Extraction*  6:**for each** $AGV_{k}\in U_{AGV}$ **do**  7:      $R_{Valid}\leftarrow \emptyset$                                                 ▹ Initialize valid multipath set for aggregate analysis  8:      $R_{Launched}\leftarrow \mathrm{SHOOT}\_\mathrm{RAYS}\left(Tx\right)$  9:      **for each** $Ray_{i}\in R_{Launched}$ **do**10:            $Path_{i}\leftarrow \mathrm{TRACE}\_\mathrm{GEOMETRIC}\_\mathrm{TRAJECTORY}\left(Ray_{i},E_{Twin},N_{refl}\right)$11:            $P_{rx,i}\leftarrow \mathrm{COMPUTE}\_\mathrm{ANALYTICAL}\_\mathrm{CORRECTION}\left(Path_{i},{\mathrm{FSPL}}_{140GHz},{\mathrm{Absorption}}_{Mat}\right)$12:            $R_{Valid}\leftarrow R_{Valid}\cup \left\{Path_{i}\right\}$                                         ▹ Retain all paths up to $N_{refl}=3$13:      **end for**    *// Compute Aggregate Spatial-Temporal Metrics for $AGV_{k}$*14:      $PL_{CI}\left(k\right)\leftarrow \mathrm{CALCULATE}\_\mathrm{CLOSE}\_\mathrm{IN}\_\mathrm{PATH}\_\mathrm{LOSS}\left(R_{Valid}\right)$15:      $P_{r}\left(k\right)\leftarrow \mathrm{COMPUTE}\_\mathrm{TOTAL}\_\mathrm{RECEIVED}\_\mathrm{POWER}\left(R_{Valid},Pattern_{3D}\right)$                    ▹ Aggregate wideband power16:      **if** $P_{r}\left(k\right)<P_{sens}$ **then**17:          Outage(k)←TRUE                                   ▹ System outage evaluated strictly on aggregate power18:      **else**19:          Outage(k)←FALSE20:          $\tau_{RMS}\left(k\right)\leftarrow \mathrm{COMPUTE}\_\mathrm{RMS}\_\mathrm{DELAY}\_\mathrm{SPREAD}\left(R_{Valid}\right)$21:          $\left[\sigma_{AoA}\left(k\right),\sigma_{AoD}\left(k\right)\right]\leftarrow \mathrm{COMPUTE}\_\mathrm{ANGULAR}\_\mathrm{SPREADS}\left(R_{Valid}\right)$22:      **end if**23:**end for**24:**return** $PL_{CI},P_{r},\tau_{RMS},\sigma_{AoA},\sigma_{AoD},Outage$

## 5. Results and Discussions

This segment presents the propagation features of the 140 GHz band for the specific region through the deterministic 3D SBR (shooting and bouncing rays) ray-tracing simulation technique. In this case, the realistic radiation features of a specially designed 4 × 4 antenna array located within an industrial digital twin setting have been analyzed. From the outcomes of the analysis, there is a systematic assessment of the large-scale coverage, temporal propagation, and spatial angular channel features. There are important implications from these outcomes for the physical-layer of 6G URLLC networks.

### 5.1. Electromagnetic Performance and Radiation Characteristics of the 140 GHz Antenna

Figure 5 below shows the reflection coefficient ($S_{11}$) of the designed antenna. In this case, the operational bandwidth of the antenna is around 6 GHz, and the antenna exhibits only one resonant frequency at 140.40 GHz. At the resonant frequency, the $S_{11}$ reflection coefficient value is −16.86 dB. This reflection coefficient value in the $S_{11}$ parameter implies that the impedance matching of the antenna is very good at the desired sub-THz resonant frequency. In this way, power losses are minimized by the antenna at this frequency, and radiation becomes highly effective. The main objective of the designed antenna is to improve the operating bandwidth of the antenna in the D-band. From the results, it can be seen that this objective has been fulfilled effectively. The appropriate wideband behavior, along with the resonance, enables the antenna to be used in 6G Industrial IoT applications that require broadband high-frequency coverage.

Two-dimensional radiation characteristics of the designed antenna element in the far-field region at 140 GHz are given in Figure 6 and provide a comprehensive understanding of the energy distribution of the designed antenna in the elevation plane (Phi = 90°). Below is a diagram that will provide useful data about the directional characteristics of the antenna, as it explains how the antenna radiates its energy in specific spatial directions. When considering the radiation characteristics of the proposed antenna at the resonant frequency, it is clearly seen that it has highly directional radiation properties. Namely, the maximum magnitude of the realized gain of the main lobe occurs at 6.74 dBi at 17.0 degrees. Such a property is absolutely preferred in sub-THz devices due to overcoming the high free-space path loss with the directed energy instead of omnidirectional emission of energy. Besides, it has 91.4 degrees 3 dB angular width, which means a sufficiently wide coverage area for a single radiating element. The side lobe level can be adequately controlled at −12.9 dB in order to suppress any backward energy leakage. The strength of the directional feature makes sure that the single antenna element performs optimally, providing the high-gain basis required for the next step of 4 × 4 array synthesis in the smart factory.

Figure 7 provides the 3D realized gain for the designed 4x4 antenna array operating at 140 GHz, thereby giving insight into various spatial performance parameters. While moving from the single patch element to the entire array design, the use of the Active Element Pattern approach was made to effectively factor in the effects of mutual coupling and embedding features in the array structure. The gain parameter is crucial to assessing the performance of the array as far as its ability to transmit/receive sub-THz signals in its intended industrial application scenario is concerned. The gain obtained in this array design, having an outstanding maximum realized gain value of 16.86 dBi, shows the extent to which the array can boost the signal power in a certain direction compared to an ideal antenna that radiates equal power in all directions. Directivity, on the other hand, measures how much the antenna’s radiation pattern is focused to form a narrow pencil beam. It shows how much the 4 × 4 array concentrates its energy in a particular direction in order to counteract the heavy path loss that occurs at 140 GHz free space.

### 5.2. Spatial-Temporal Channel Characterization and Propagation Metrics

The plots of spatial distributions of the SNR and channel capacity for a deterministic ray tracing simulation model of a 140 GHz access point in the dense smart factory environment are depicted in Figure 8. Figure 8 is composed of two plots located next to each other. These plots depict the SNR distribution in dB and the Achieved Channel Capacity in Gbps for the designed 4 × 4 directional antenna array, from left to right, respectively. MATLAB model of the industry included the consideration of the highly directional 3D radiation pattern of the microstrip patch antenna array along with the multipath reflections obtained from the SBR method. The color scale represents SNR values between −60 dB and approximately 15 dB in the left image, and channel capacity values ranging from 0 to 9 Gbps in the right image. Contrary to conventional isotropic setups, the coverage area in the factory environment clearly demonstrates the spatial filtering of the array, giving rise to a unique cross-shaped propagation pattern governed by the main and side lobes. Within the beams where the communication channel is targeted, the signal manages to efficiently compensate for the extremely high path loss at 140 GHz frequency, which enables highly focused coverage and thus ensures maximum channel capacities of up to 9 Gbps. However, outside of these precisely targeted directional beams, extreme attenuation and isolation effects at the sub-THz band lead to almost zero capacity in those areas not lit by the beams, affecting the positioning of the AGVs and the overall design of the 6G IIoT network itself.

Graphs given in Figure 9 show the propagation behavior of the 140 GHz sub-THz channel in the smart factory scenario. Figure 9 has two sub-plots, which are Figure 9a Path Loss vs. Distance, and Figure 9b Cumulative Distribution Function (CDF) of the Received Wideband Power. The Figure 9a has a horizontal x-axis that has the linear distance in meters, and the vertical y-axis shows the path loss in dB. In the scatter plot (blue dots) in the Figure 9a, deterministic path losses are shown through the SBR ray-tracing algorithm with the multipath effect, while the dashed red line in the graph represents the Free Space Path Loss (FSPL) theoretical baseline. This graph helps in better understanding of the attenuation due to metallic industrial clutter in the short range of distances (4 to 12 m). From the graph, it is observed that although the deterministic multipath path loss follows the pattern of FSPL, there are severe spikes in attenuation at certain points (such as exceeding 105 dB at approximately 10 m). These outliers represent the presence of severe NLoS due to blockage from CNC machines. Figure 9b shows the cumulative distribution function (CDF) of received wideband power at distinct AGV positions, where the x-axis is in dBm and the y-axis is the probability. The received power is computed based on the transmitter power (10 dBm), the gain of the 4 × 4 directional antenna array, extreme sub-THz path loss, and metallic reflections, completely omitting any outdoor factors such as atmospheric and vegetation losses. Due to the isotropic nature of the receiver, the extracted value of received power, and the zero-outage performance in the targeted operational sector define the worst possible scenario. With the implementation of 6G physical-layer technology, a directional antenna of an AGV will contribute even more array gain to the received power. It is clear from CDF that most of the received power values on the spatial grid fall in the range of −65 dBm to −47 dBm. It can therefore be concluded that regardless of the extreme NLoS obstructions and the highly attenuated nature of the 140 GHz carrier frequency, the highly directional antenna arrangement ensures high signal reception levels, far above the −90 dBm sensitivity level.

Figure 10 shows the Power Delay Profile (PDP) and the Cumulative Distribution Function (CDF) of the RMS delay spread for the 140 GHz carrier frequency in the smart factory environment. As the propagation delay increases, the received signal power of the multipath components decreases significantly, as depicted in Figure 10a. This reduction is due to the path loss at the sub-THz level as well as the many specular reflections that result from the increased geometrical communication range. This is clearly evident from the graph, where early-arriving components (denoting LoS path or dominant first order reflections) have a considerably greater signal strength than late-arriving NLoS components. This indicates that the spatial filtering capability of the highly directional antenna array plays a vital role in ensuring transmission efficiency in 6G IIoT systems. Particularly at the 140 GHz frequency, the received signal strength levels of the highly delayed NLoS components (approaching 140 ns) drop rapidly to values very close to the −90 dBm sensitivity threshold. This automatically excludes multipath interference with a long tail. It is evident from the outcome of the analysis that the use of directional antennas, and metallic environments is a critical factor affecting the performance of 6G mobile communication systems over time. As demonstrated in Figure 10b, the RMS delay spread has a tight bound and can occur within 10 ns and 16 ns with a very high probability. Such a small temporal dispersion is an overwhelming benefit rather than an issue. This underscores the significance of deterministic ray tracing-based channel modeling under highly cluttered factory indoor conditions where the NLoS conditions and metal reflections are prevalent. This underscores the fact that such an extremely low delay spread enables physical-layer of URLLC requirements and ensures sustainable communication without channel equalizers in such environments. Channel modeling, which takes into account not only the distance but also the directional filters and the type of industrial clutter, is an important requirement.

Figure 11 depicts the angular properties of the propagation obtained for the 140 GHz fixed access point located inside the highly cluttered smart factory environment. To properly examine the spatial scattering, and due to the three-dimensional (3D) propagation environment, the spatial metrics are separately examined in the azimuth and elevation planes for both the Angle of Departure (AoD) and Angle of Arrival (AoA).

The AoD distributions at the transmitter side, shown in Figure 11a,b, are characterized by very high spatial confinement. Specifically, the Azimuth AoD (Figure 11a) has a very narrowly distributed main departure beam at 270°, accompanied by another narrow departure beam at 180°. In addition, the Elevation AoD (Figure 11b) has very narrow elevation confinement, in that all surviving beams depart from close to 0° on the horizontal axis. This extremely narrow 3D departure distribution proves the physical realization of very stringent spatial filtering through the highly directional 4 × 4 antenna array. Subsequently, the transmitted sub-THz energy is very well contained within the confines of the beamwidth of the antenna array, hence starving physically unrealistic multipath propagation paths before engaging with the surroundings. In contrast, Figure 11c,d show the incoming angular spreads at the AGV receiver, capturing the significant scattering effect of the metallic environmental components such as CNC machines and racks. In the case of Azimuth AoA (Figure 11c), multipath-rich clusters appear. The presence of paths that arrive from completely opposite quadrants, i.e., the clusters at 90°, 180°, and 270° suggests the dominance of multi-bounce reflections in the horizontal plane around the AGV. In the case of Elevation AoA (Figure 11d), although somewhat spread-out compared to its departure counterpart because of scattering from local clutter, the cluster is mostly confined close to the 0° elevation plane, owing to the plane-wave propagation in the factory floor. The clear spatial asymmetry between the highly localized transmitter (AoD) and the scattered receiver (AoA) underscores the spatial diversity of the industrial channel. Derivation of such distributions is therefore an essential source of knowledge for designing reliable 6G Massive MIMO and beam tracking systems in highly reflective environments.

Table 5 shows an extensive benchmarking of the achieved 140 GHz channel parameters in the spatial-temporal domain with respect to the empirical results achieved at 142 GHz frequency from [9]. A notable point with regard to the temporal dispersion is that our framework produces RMS delay spreads from 9.8 ns in LoS conditions to 21.5 ns in deep NLoS situations. While these figures might seem a bit higher in comparison with the 7.4 ns to 9.7 ns range stated in the reference work, this is physically consistent due to the fact of multiple reflections of signals resulting from a high concentration of metal objects in our virtual representation of the smart factory. In addition, there is a huge physical difference found in the spatial measurements, which confirms our approach to the hardware-software integration. Calculated through power-weighted circular statistics, the highly concentrated Azimuth AoD (2.0° to 6.5°) in the designed architecture is perfectly correlated with the horizontal angles of [9] (2.3° to 5.1°), whereas the elevation range of it is physically constrained below 2.0° This mathematical calculation proves the efficiency of the designed 4 × 4 transmitter array in spatial filtering. However, the azimuth angle of AoA in the proposed architecture is highly wider (82.9° in deep NLoS versus 6.3 in [9]). The reason behind this high angular distribution is due to the spatial scattering effect from the surrounding industrial machines at the receiver end. Lastly, despite encountering a very high path loss of 106.5 dB and angular distribution in the deep NLoS scenario, the designed architecture provides a zero outage performance at the targeted operating point with −64.7 dBm received power.

In order to fully separate and measure the physical effect of the spatial filtering technique, an ablation experiment was conducted in the digital twin, as described in Table 6. As part of this experiment, propagation and the Effective Isotropic Radiated Power (EIRP) were kept constant and equal to 26.86 dBm for both the transmitter configurations. If the isotropic antenna (0 dBi) is used as the transmitter, then the spherical propagation of radio waves fully covers the metallic obstacles in the factory floor environment. The absence of spatial filtering leads to a large number of multipath reflections of a higher order, which, in turn, produces a high delay spread greater than 120 ns with a very dispersive AoA pattern (∼102.4°). In contrast, the use of the suggested AEP synthesis for the 4 × 4 directional array fundamentally changes the principles of propagation. The extremely focused main lobe (16.86 dBi maximum gain) imposes a strict restriction on the AoD, starving all redundant geometrical pathways of any energy prior to the interaction with the peripheral CNC equipment. As a result of this physical process, the harsh multi-reflections become automatically suppressed, thus minimizing the maximum RMS delay spread to 21.5 ns. Moreover, when the differences have been established, it is important to look into the effects on basic communication parameters like effective power received and channel capacity in identical normalized environments. In spite of the fact that a very high power injection (26.86 dBm Tx Power) is required for an isotropic environment to make the raw aggregate power of transmission similar to that of the directional antenna array, the communication suffers catastrophically. This particular >120ns delay spread leads to significant frequency-selective fading and results in ISI that far outperforms the cyclic prefix in standard sub-THz OFDM systems. Thus, under these very same EIRP parameters, the effective capacity of the isotropic configuration reduces to zero for URLLC due to the inability to perform equalization. The spatial filtering provided by the 4 × 4 configuration, however, enables high-capacity links. In summary, this research illustrates the importance of taking into account the AEP-based synthesized 3D radiation pattern in order to consider the effect of spatial filtering and avoid underestimating temporal dispersion and the risks of ISI under certain waveform numerologies in the sub-THz factory scenarios.

In conclusion, the results show the potential of the developed channel model in precisely controlling the RMS delay spread while maintaining a resilient zero outage within the targeted operational sector. Thus, our deterministic model offers an extremely promising physical layer basis for integration into future 6G industrial wireless networks, where both low latency and robust coverage are equally important. The combination of high-precision 3D SBR ray tracing technique and a specifically designed highly directional antenna array allows the mitigation of significant temporal dispersion in metallic smart factories.

In order to establish the necessary spectral context for the high-fidelity deterministic simulation model, Table 7 below gives an extensive review of existing propagation studies. It is essential to reiterate that the goal of this comparative analysis between papers is only to place the work into proper spectral context and not to claim any methodological superiority. Any quantitative comparison of performance across these studies is not possible on a methodological level because all of these systems have very different physical boundaries in terms of environment, antenna setup, transmit power, receiver threshold, and bandwidth. Rather, Table 7 provides an example of physical scaling of propagation phenomena in the literature from sub-6GHz and mmWave networks to the 300 GHz THz band. Through such comparative analysis, it becomes clear that the 140 GHz directional model proposed by this paper fits the time-space requirements of future networks without overstating multi-bounce scattering.

### 5.3. Limitations and Theoretical Bias of Specular Assumptions

With respect to the 140 GHz sub-THz band, the extremely short wavelength of about 2.14 mm means that very fine surface roughness of industrial machines can easily exceed the Rayleigh criterion. The existing digital twin, which is based on the deterministic approach, assumes that the interior metallic objects represent the perfectly conducting specular surfaces. The basic assumption of the theory that is evident in practical terms is in the Power Delay Profile (PDP). Because of geometric reflections only, the SBR engine generates highly discrete impulse arrivals. However, in a real-life metallic smart factory, physical diffraction will lead to smearing of these impulse arrivals due to the fact that the energy from specular reflection will be transferred to the diffuse multipath component. This fact means that the proposed model tends to overestimate the power amplitude of the main NLoS arrivals and underestimate the density of the multipath components.

Such an approach to idealization brings about the formation of a theoretical bias in the obtained values of the spatial dispersion, which is called the Azimuth Spread of Arrival (ASA). The spatial dispersion in the considered model, which reaches up to 82.9° in deep NLoS conditions, is caused by macro-reflections, which happen due to strict compliance with Snell’s law. However, the real nature of surfaces gives rise to an expansion of the angular range, since the electromagnetic energy emitted by a particular surface is scattered in various directions, rather than being concentrated in one. Thus, the real angular spread in the receiver will be somewhat larger and more homogeneous in space than the considered deterministic angular spreads.

Above all, the lack of a diffuse scattering model means a conservative nature for the NLoS coverage predictions. As a matter of fact, a pure specular ray-tracing algorithm will always produce sharp shadows, which would mean the existence of dark regions behind the metallic obstacles having no capacity at all. Diffuse scattering in the real world serves as an ambient spatial distribution scheme that constantly redistributes the leftover scattered signals towards the shaded areas. Although specular rays have scattering losses when reflecting off rough surfaces, the diffused phenomenon ensures the illumination of the shadowed area where no specular ray can reach, mathematically speaking. Consequently, the capacity mapping and zero outage coverage results in this work can be considered as a baseline scenario; in reality, NLoS spatial coverage may well have some room for improvement due to this diffuse ambient effect.

### 5.4. Trade-Off Analysis and Composite Figure of Merit

The use of highly directional antenna arrays operating at 140 GHz becomes a physical necessity to compensate for high free-space path losses and temporal dispersion. Yet, such physical benefits necessarily come with costs in the form of decreased coverage area, increased sensitivity to beam alignment, and increased complexity of beam management. It can be difficult to estimate the actual physical cost of sub-THz networks operating in an environment with high levels of cluttering when studying physical-layer characteristics separately.

Based on the joint performance-complexity approach for the evaluation of wireless systems [51], we propose a new normalized Composite Figure of Merit (Φ) that allows us to quantitatively characterize the tradeoff. The newly proposed FoM is formulated as a mathematical balance between communication gains at the physical layer and disadvantages due to narrow beamwidths.

(15) $$\Phi =w_{c}\cdot \hat{C}-w_{\tau }\cdot {\hat{\tau }}_{RMS}-w_{out}\cdot P_{outage}-w_{align}\cdot \Psi_{align}$$

where, $\hat{C}$ stands for the normalized capacity of the target sector (benefit term). The following parameters stand for the penalty terms: ${\hat{\tau }}_{RMS}$ stands for the normalized RMS delay spread, $P_{outage}$ stands for the spatial outage probability beyond the targeted sector, while $\Psi_{align}$ stands for the beam alignment vulnerability. It is very important to state that $\Psi_{align}$ is totally controlled by the physical limitations of the hardware; in particular, it is inversely proportional to the half power beamwidth (HPBW) of the antenna, not channel angular spread. The parameters $w_{c}$, $w_{\tau }$, $w_{out}$ and $w_{align}$ are the application-dependent normalized weights ($\sum w_{i}=1$) of the operation priorities.

In order to have the quantified results on the sensitivity of the above formulation, the normalized Figure of Merit (Φ) of the Isotropic vs. 4 × 4 array scenarios from Table 6 is analyzed under two different weight settings of operation. For the quantified evaluation, the variables are scaled to the [0,1] range. While the 4 × 4 array provides high capacity ($\hat{C}\approx 1.0$) and low delay spread (${\hat{\tau }}_{RMS}\approx 0.1$), it experiences heavy penalties in terms of outage ($P_{outage}\approx 0.85$) and alignment ($\Psi_{align}\approx 0.9$ as a result of the narrow HPBW). On the other hand, the isotropic antenna has no penalty in the spatial aspect ($P_{outage}\approx 0,\Psi_{align}\approx 0$), but a heavy penalty in temporal spread (${\hat{\tau }}_{RMS}\approx 1.0$) and sectoral capacity ($\hat{C}\approx 0.1$).

Scenario A (URLLC-oriented): Emphasis on capacity and low latency ($w_{c}=0.45,w_{\tau }=0.45,w_{out}=0.05,w_{align}=0.05$). In this case, the FoM (Φ) score for the 4 × 4 array is 0.31, whereas for isotropic, it results in −0.40. Clearly, the directional antenna array outperforms.

Scenario B (Broad Coverage/IoT): With emphasis on spatial robustness and alignment simplicity ($w_{c}=0.1$, $w_{\tau }=0.1$, $w_{out}=0.4$, $w_{align}=0.4$). At such weightage values, the FoM value of the 4 × 4 array reduces to −0.61, while the isotropic configuration gets to −0.09.

This measurable sensitivity analysis clearly shows that this switch to more directional 6G sub-THz regimes is only useful under certain operating conditions, and will require new beam tracking strategies to account for the significant physical layer spatial losses.

## 6. Conclusions

For the current work, a geometry-based characterization of the sub-THz channel at 140 GHz has been conducted with a deterministic industrial digital twin with considerable success. The multipath channel environment, along with the AEP-based synthesized 3D radiation pattern of the custom 4 × 4 microstrip patch antenna array, has been accurately modeled by employing the pattern multiplication technique. The simulation model developed using the combination of full-wave electromagnetic design and geometric optics has been used to simulate the multipath conditions at 30 different AGV locations when extreme NLoS conditions exist, for which the traditional isotropic assumption is not applicable. High-fidelity deterministic simulation model was ensured by the extraction of the sub-THz channel characteristics by employing the deterministic ray-tracing model with 3-reflections SBR method, with analytical correction to 140 GHz free-space and material absorption effects. In such stringent conditions of simulation, it was evident from the numerical calculations that even when the AGVs faced extreme NLoS attenuation that reached 106.5 dB, along with a large azimuth angle of arrival spread up to 82.9° due to metallic scattering, the proposed scheme worked perfectly fine by ensuring zero outages in the targeted operational sector. This is facilitated by the fact that spatial filtering of the 4 × 4 antenna array ensures that the azimuth angle of departure spread to 6.5°. Consequently, directional beamforming automatically eliminates multi-path reflections, limiting the maximum value of the RMS delay spread to a relatively small value of 21.5 ns. This small temporal spread reduces the possibility of ISI as long as the delay spread stays below the cyclic prefix of the used sub-THz signal. It is evident from the results that the traditional ray tracing technique using isotropic transmitters produces unrealistically complex multipath behavior, which causes grossly exaggerated delay spread in metal clutter scenarios. Additionally, the application of realistic, highly directional antenna arrays natively acts as a spatial filter, generating an exceptionally sparse Power Delay Profile (PDP) by naturally filtering out excessive multi-bounce reflections. The proposed integrated framework demonstrates that while the extreme sub-THz attenuation inherently restricts raw spatial coverage, it drastically minimizes temporal dispersion to a few nanoseconds, thereby mitigating the risk of Inter-Symbol Interference (ISI) provided that this delay spread is accommodated by the cyclic prefix of the selected sub-THz waveform. Furthermore, this tight delay spread directly supports ultra-reliable low-latency communication (URLLC) physical-layer constraints without the need for complex, power-hungry channel equalizers. Our approach has successfully bridged the critical “Antenna Assumption Gap” in the literature, yielding significantly reliable and realistic channel statistics for 6G Industrial IoT network deployments.

The limitation of our method lies mainly in the high computational complexity of the approach. With a considerable increase in ray-tracing resolution, the number of reflections or the number of receivers, computation times may become long. Nonetheless, the problem may be solved by decomposing the industrial setting into smaller spatial sectors or by applying the pattern multiplication technique to distinguish between the extraction process of single-element electromagnetic components and the whole array calculations. In addition to that, in the vast majority of situations, computation time only becomes a problem in simulations of gigantic factories, in which case the macroscopic parts can be easily modeled using specular boundary polygons. In this way, since our deterministic approach is based on the Geometrical Optics and Electromagnetic theory, it is physically predictable and geometry-based validated. Additionally, because deterministic SBR exhaustively evaluates all valid multipath propagation trajectories over a given bounce limit, it guarantees physical rigor and absolute numerical consistency at all times. On the other hand, black-box machine learning (ML) or purely stochastic channel models lack physical correctness and cannot consistently generalize to complex, cluttered industrial environments. Therefore, our ray-tracing approach demonstrates superior high-fidelity deterministic simulation model and reliability when compared to purely empirical ML-based prediction methods.

It is essential to note that the results in this study are arrived at through an extensive simulation process. Even though there is a very realistic theory due to the incorporation of full-wave electromagnetic solver data along with material properties meeting ITU standards, there is still need for extensive measurements in active industrial plants in the future. For future research, the same scenarios will be experimentally implemented in practical smart factory setups by means of frequency domain measuring techniques employing sub-THz vector network analyzers, while the data obtained from such experiments will be compared to our digital twin simulations. This would make the practical applicability of the proposed model strong and contribute much in terms of real applications for 6G IIoT systems design. Our deterministic model is easy to implement in other environments that are equally cluttered and indoors in nature. With these complicated multipath networks, effective network planning results will be achieved in keeping with URLLC physical-layer reliability requirements and also other coverage improvement goals. However, one should pay attention to the fact that although this deterministic channel model sets required physical bounds of dispersion, the full justification of URLLC requires the conduct of link-level simulations. Further study should consider specific waveforms for 6G, reliable channel coding approaches, and accurate calculations of packet error probabilities. While the proposed approach does not explicitly model the dynamic mobility of the factory components, it inherently promotes robust network dimensioning through comprehensive static spatial modeling, which can contribute to overcoming severe NLoS blockages. However, taking into account complex modeling of moving AGVs, actively used robotic manipulators, and human body obstacle blocking becomes a relevant issue for future research that may improve the proposed methodology in terms of its effectiveness. In addition, there exist different adaptive beam steering methods to satisfy multicriteria connectivity. Future research into this topic should include the modeling of reconfigurable intelligent surfaces (RIS) and active beam tracking techniques in the ray-tracing-based modeling of industrial wireless communication links at the frequency range of 140 GHz sub-THz. Further research is needed to investigate ultra-dense access point topologies and dynamic beam alignment while considering the severe reliability constraints of industrial robots. Furthermore, interference mitigation algorithms for overlap-free spatial filtering and resource sharing in scenarios where multiple 140 GHz access points operate simultaneously on the factory floor need to be developed. Ultimately, in the context of this research, the deterministic channel characterization is discussed based on the artificial digital twin model. The results derived empirically through future sub-THz implementations shall be an inspirational and pioneering source of reference for 6G research, especially concerning our proposed approach.

## Figures and Tables

**Figure 1:**
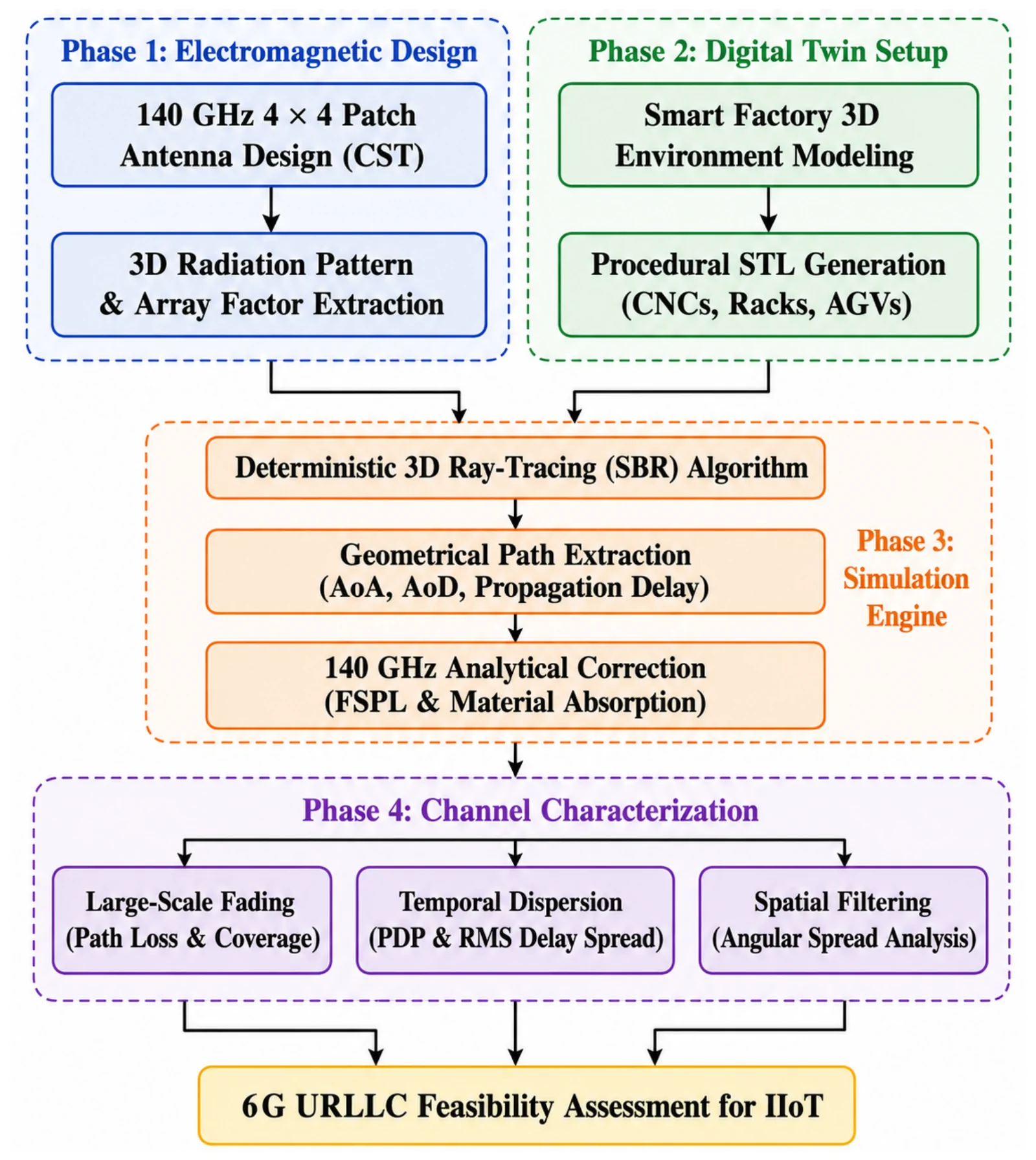
Comprehensive block diagram of the recommended deterministic channel characterization framework for 140 GHz 6G Industrial IoT (IIoT) applications.

**Figure 2:**
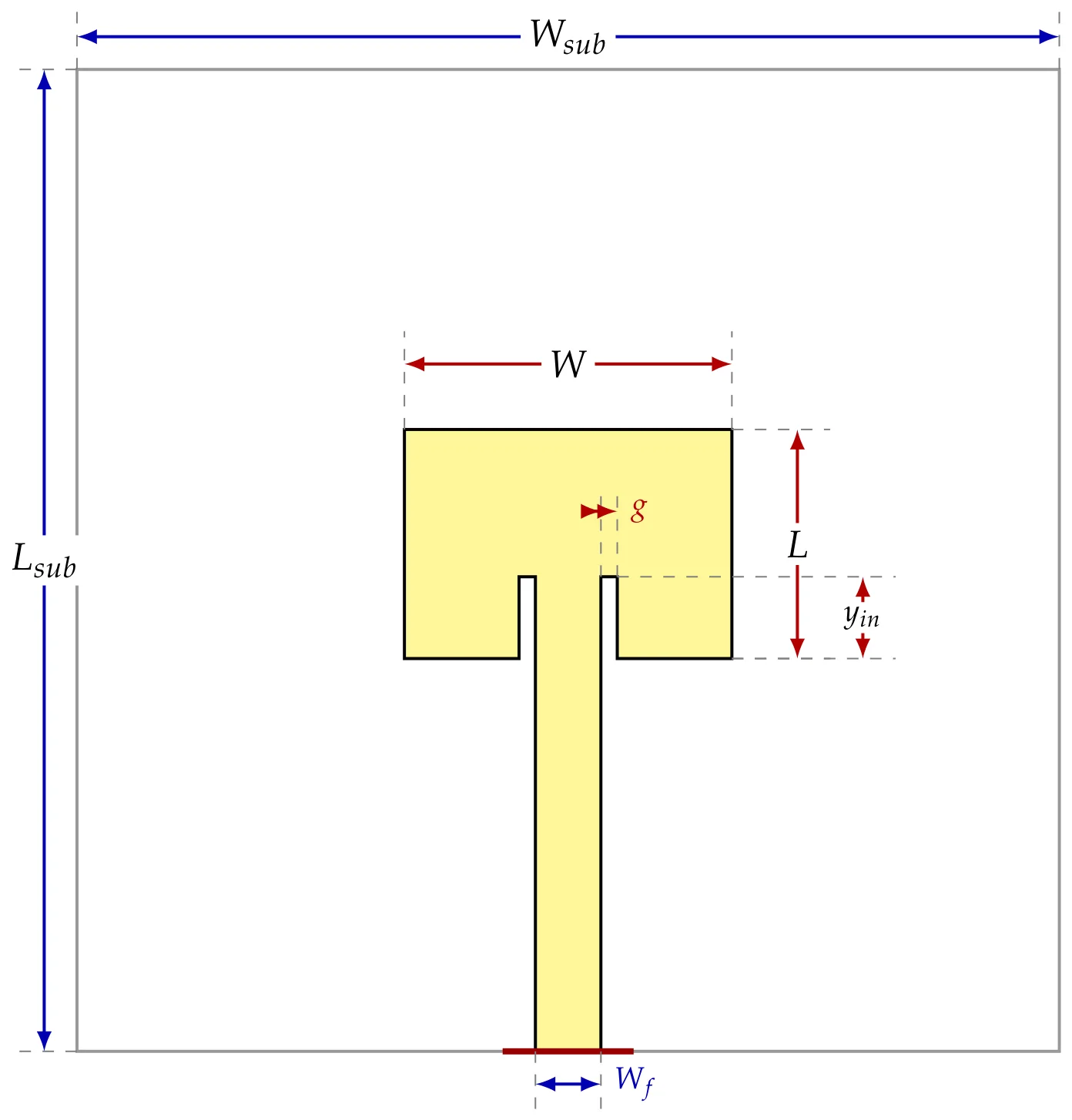
Top-view dimensioned geometric schematic of the proposed 140 GHz inset-fed microstrip patch antenna element.

**Figure 3:**
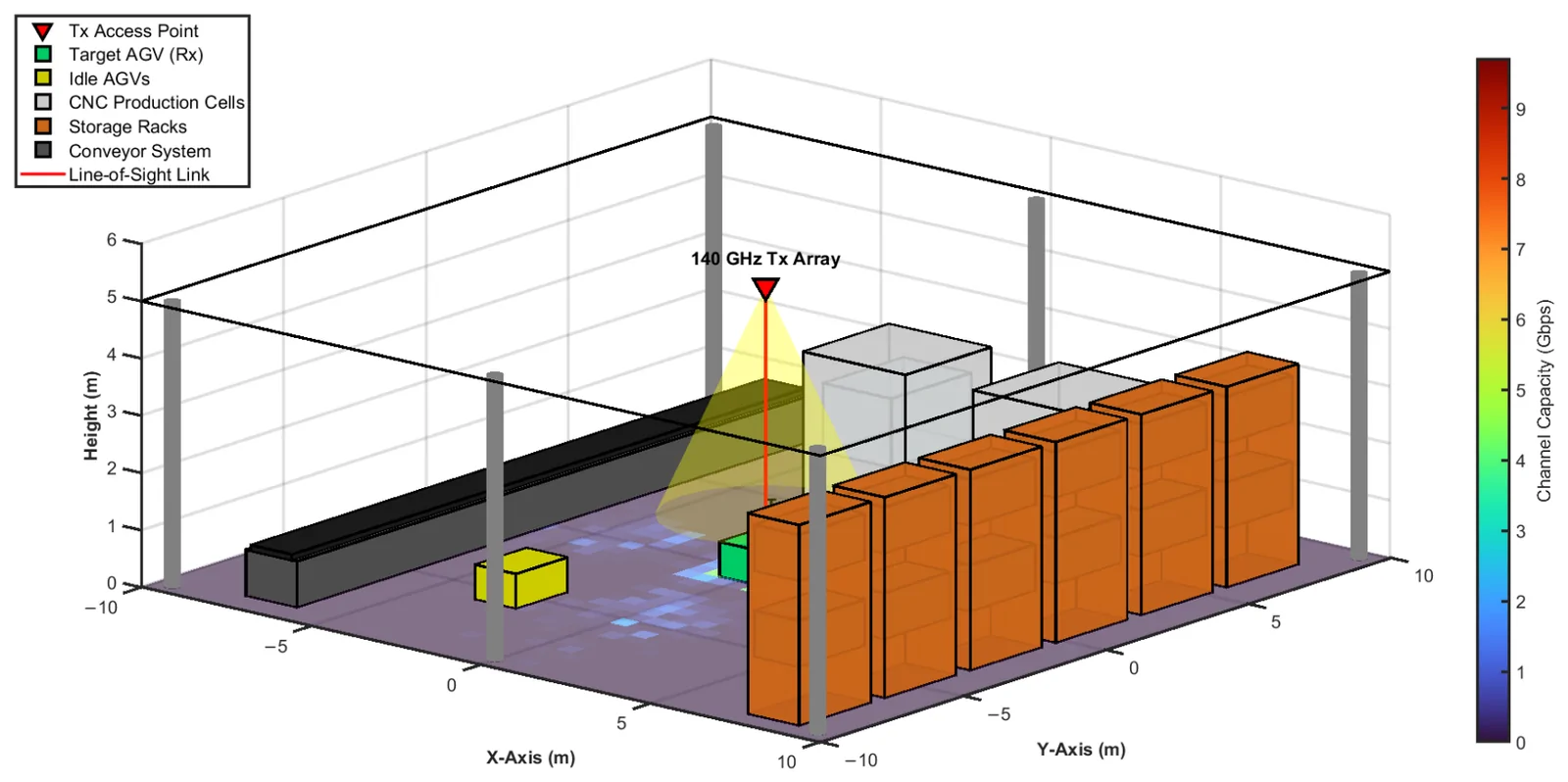
3D digital twin of the smart factory environment and the spatial heatmap of 140 GHz channel capacity.

**Figure 4:**
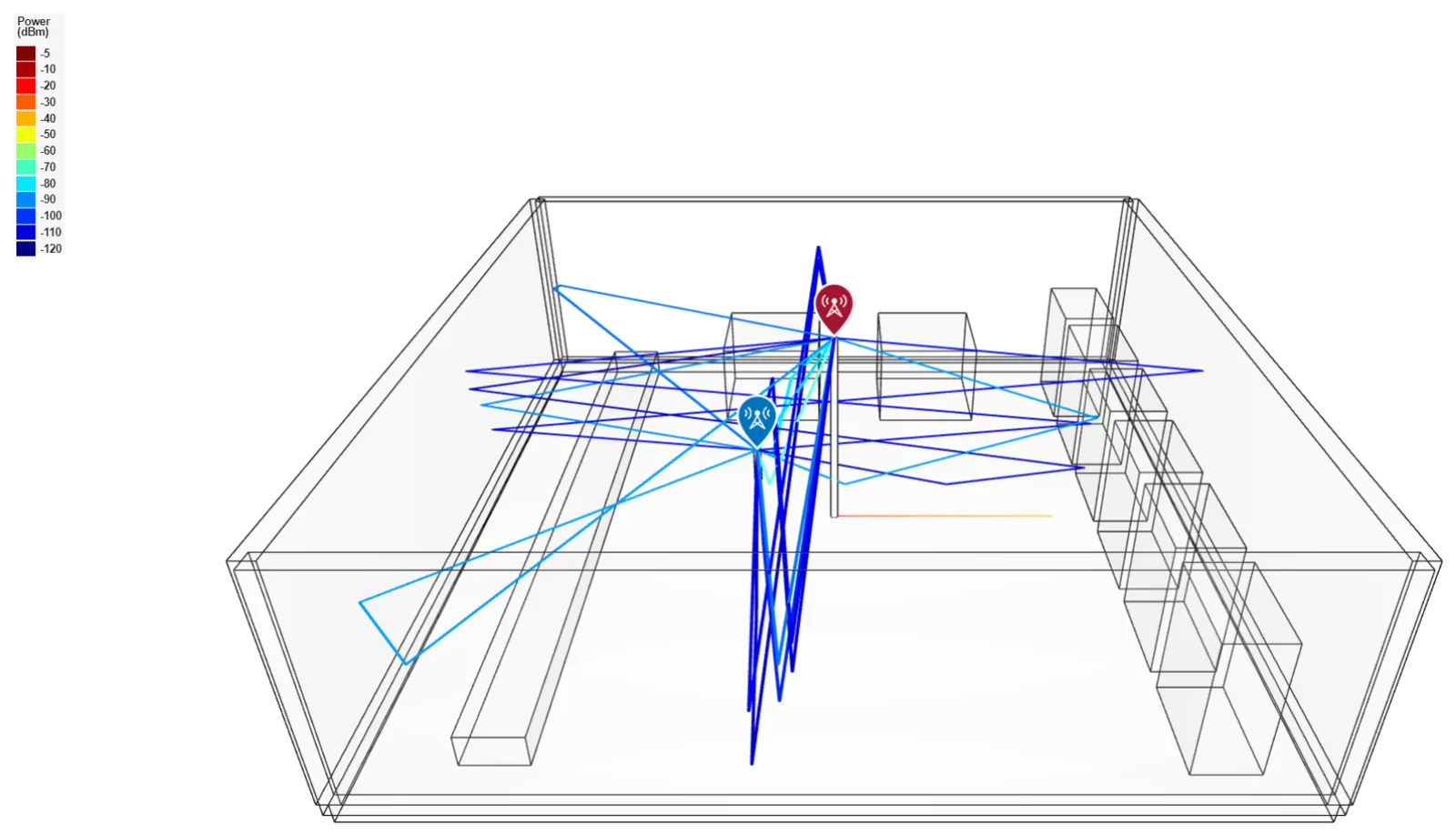
3D visualization of the deterministic SBR propagation paths within the smart factory digital twin.

**Figure 5:**
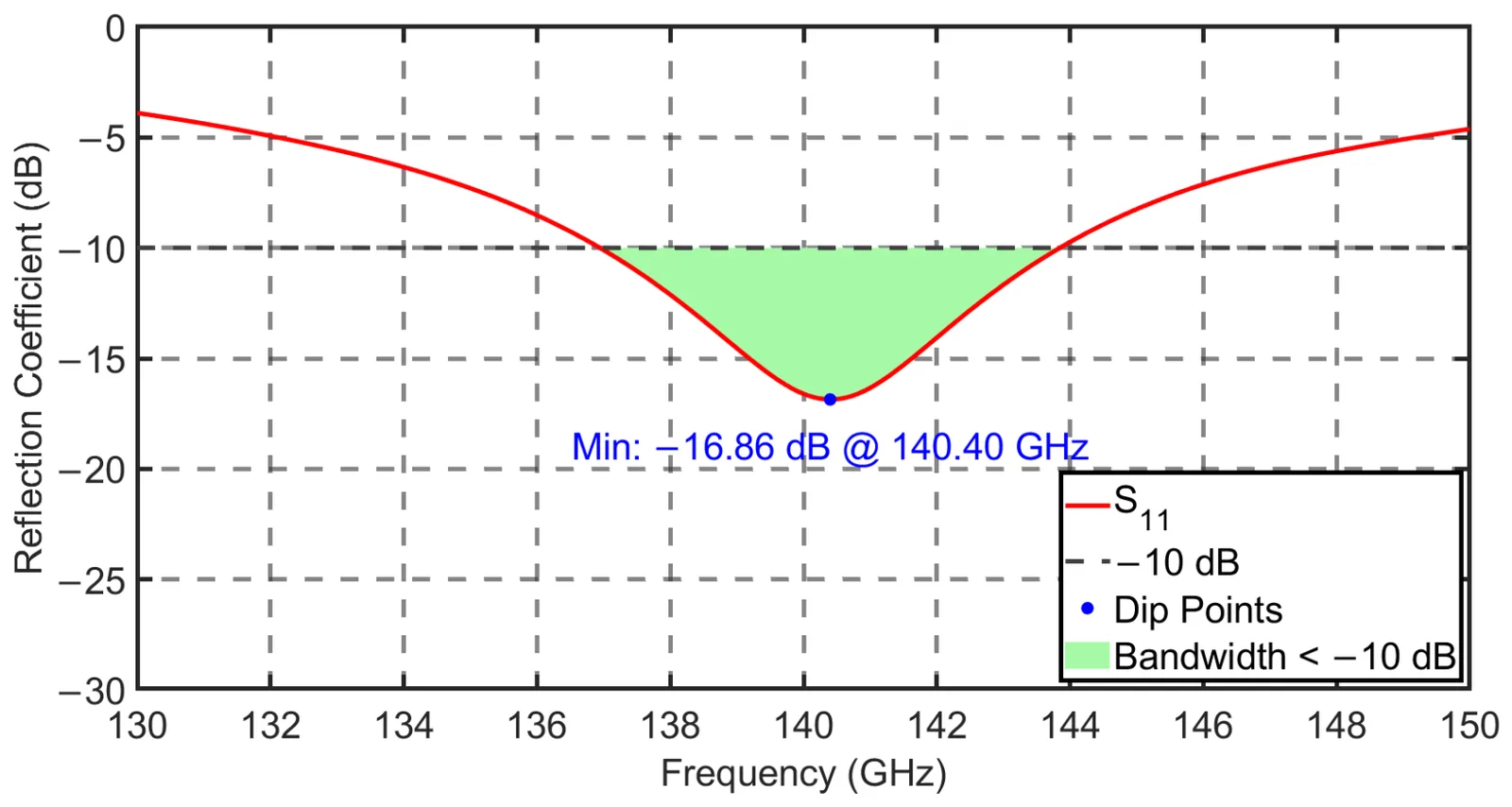
Reflection coefficient ($S_{11}$) of the suggested microstrip patch antenna element.

**Figure 6:**
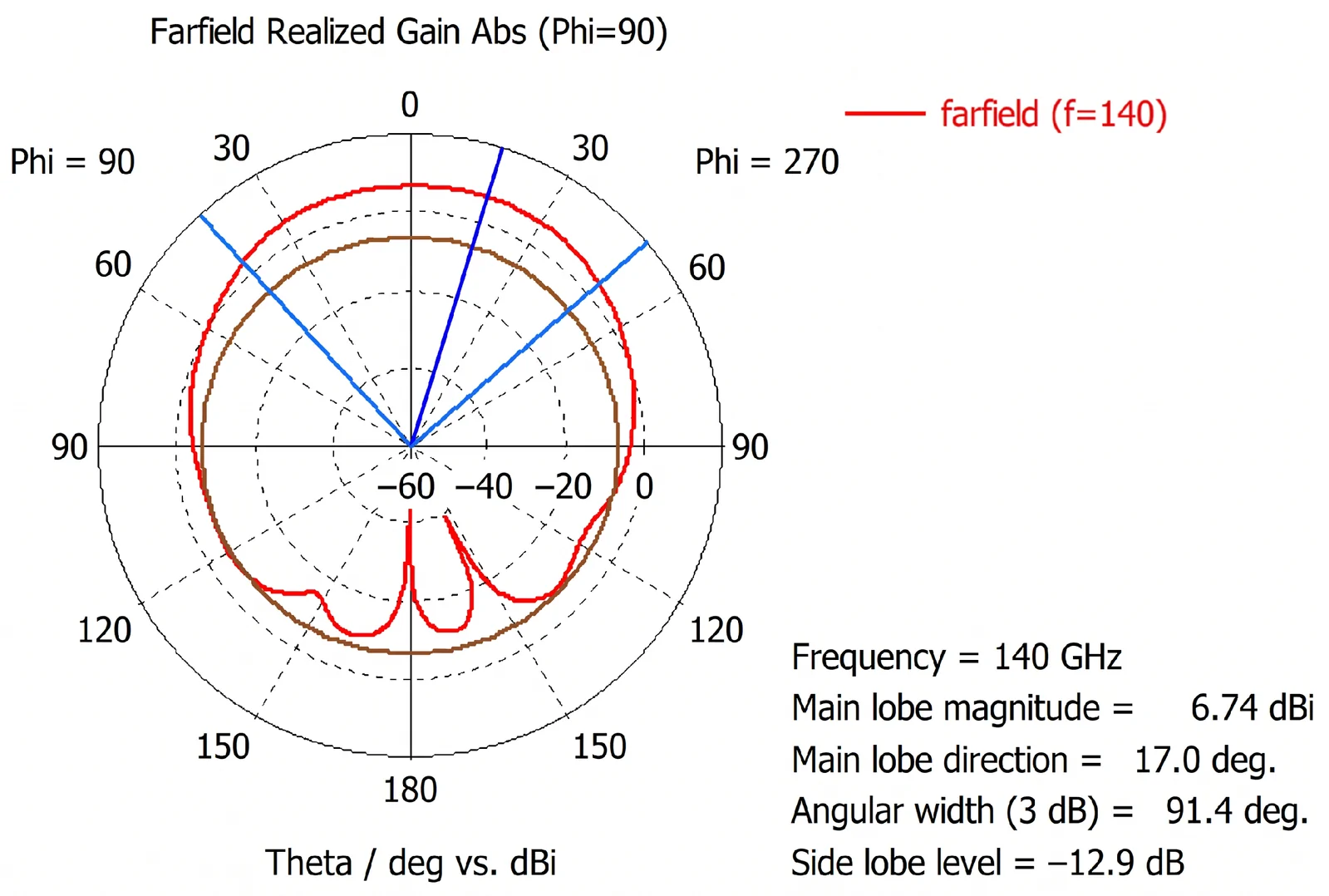
Two-dimensional far-field radiation pattern of the proposed sub-THz antenna element at 140 GHz. The red curve represents the far-field realized gain radiation pattern. The blue lines indicate the main lobe direction and the boundaries of the 3 dB angular width. The brown circle represents the side lobe level reference.

**Figure 7:**
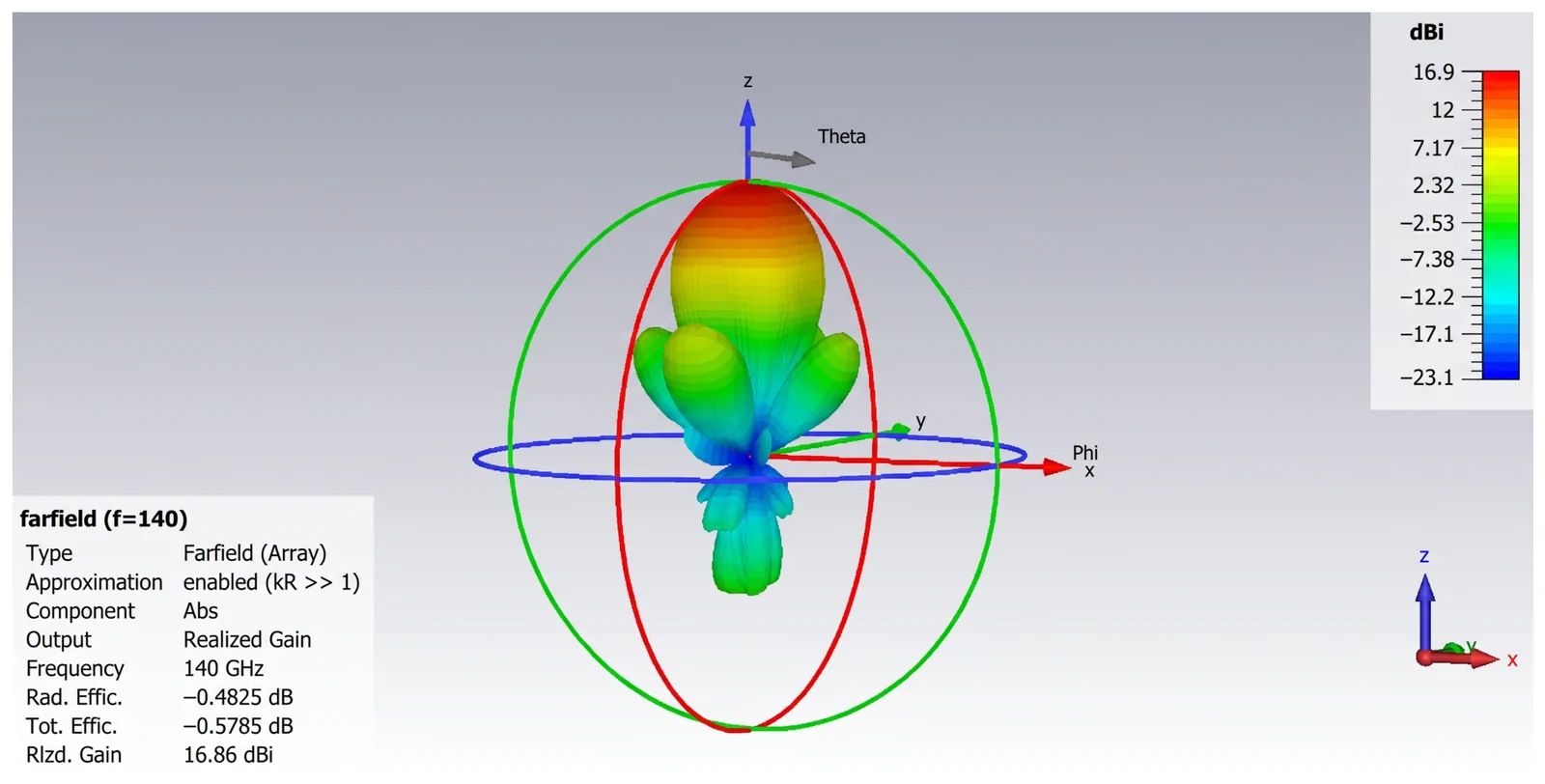
Three-dimensional far-field realized gain of the synthesized 4 × 4 microstrip patch antenna array at 140 GHz. The arrows in the 3D plot indicate the Cartesian coordinate system axes x, y, z and the spherical coordinate angles Theta, Phi.

**Figure 8:**
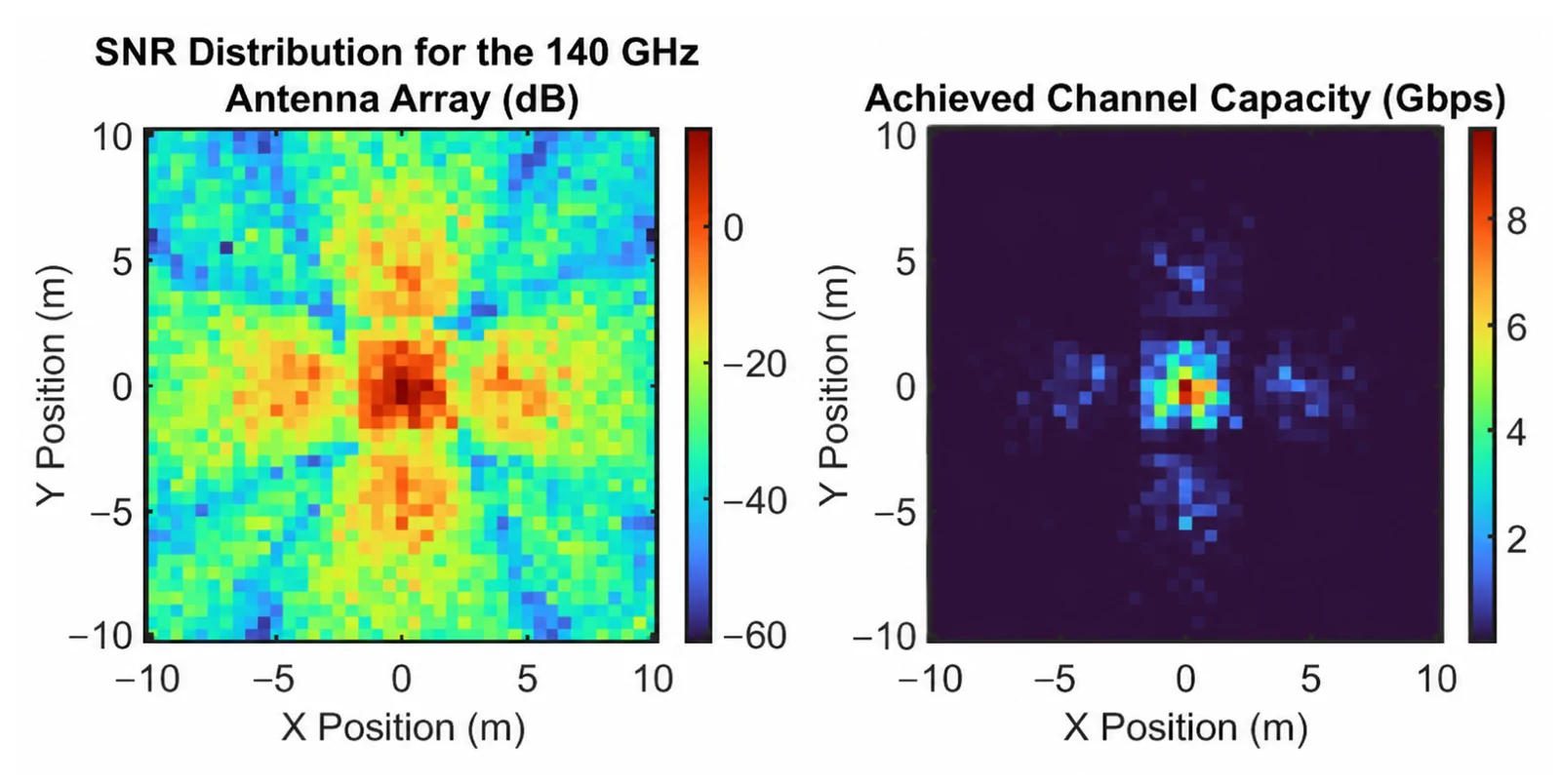
Spatial distribution of the Signal-to-Noise Ratio (SNR) in dB (**left**) and the corresponding achieved channel capacity in Gbps (**right**).

**Figure 9:**
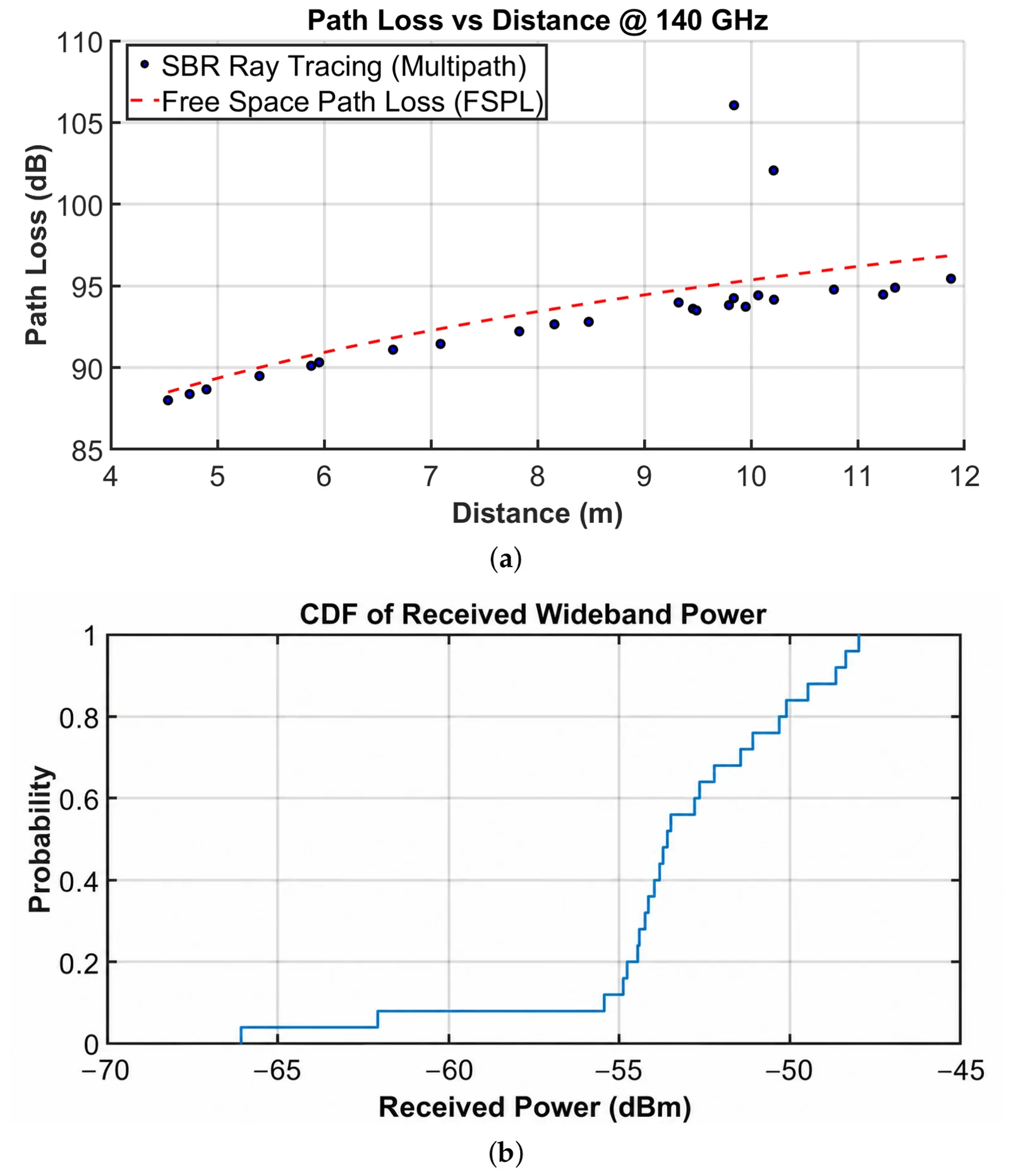
(**a**) Path loss vs. distance comparing the deterministic SBR ray-tracing results with the theoretical Free Space Path Loss (FSPL). (**b**) Cumulative Distribution Function (CDF) of the received wideband power across the autonomous guided vehicle (AGV) locations.

**Figure 10:**
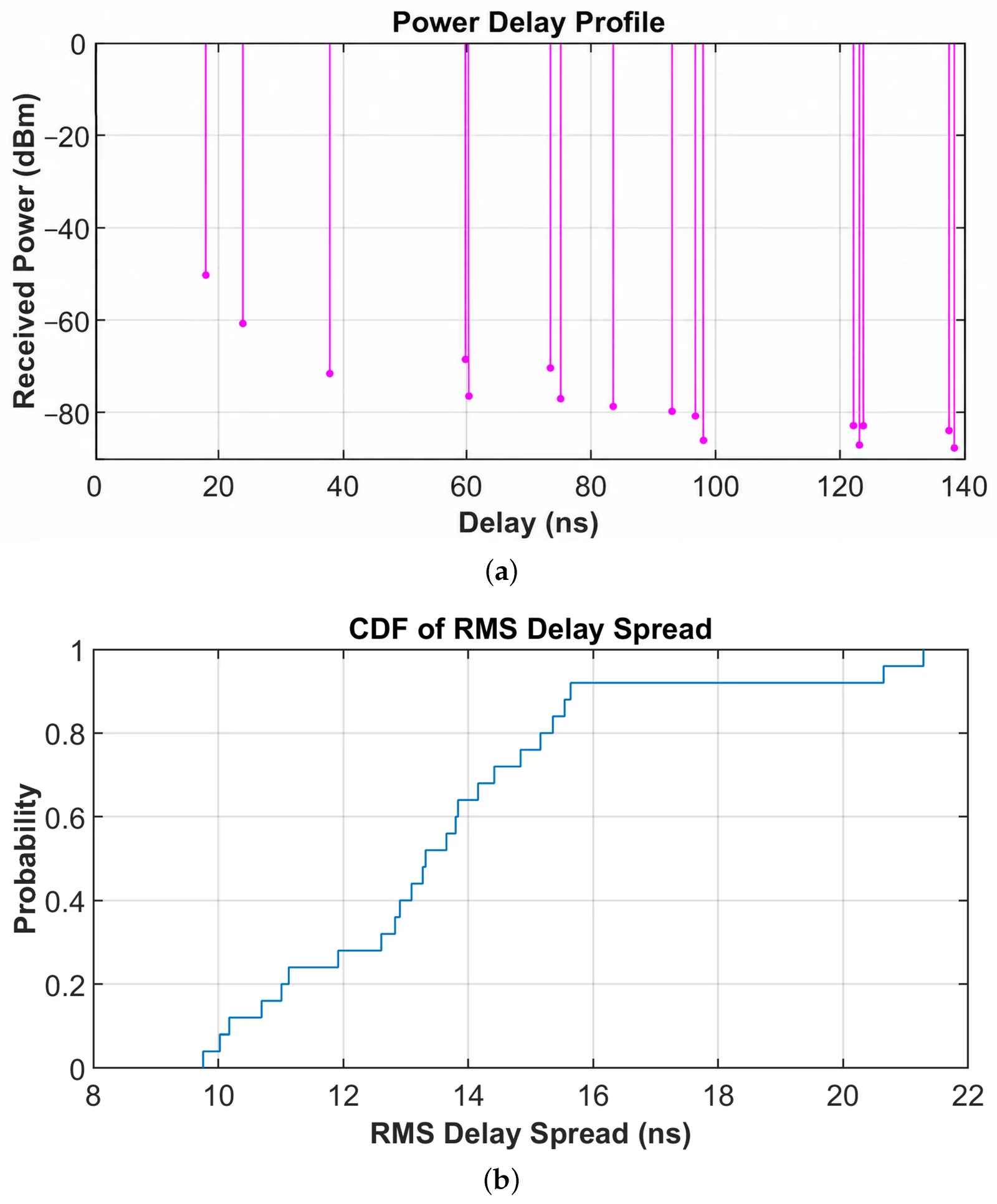
Temporal dispersion analysis of the 140 GHz channel in the smart factory digital twin. (**a**) Power Delay Profile (PDP). (**b**) CDF of the RMS delay spread.

**Figure 11:**
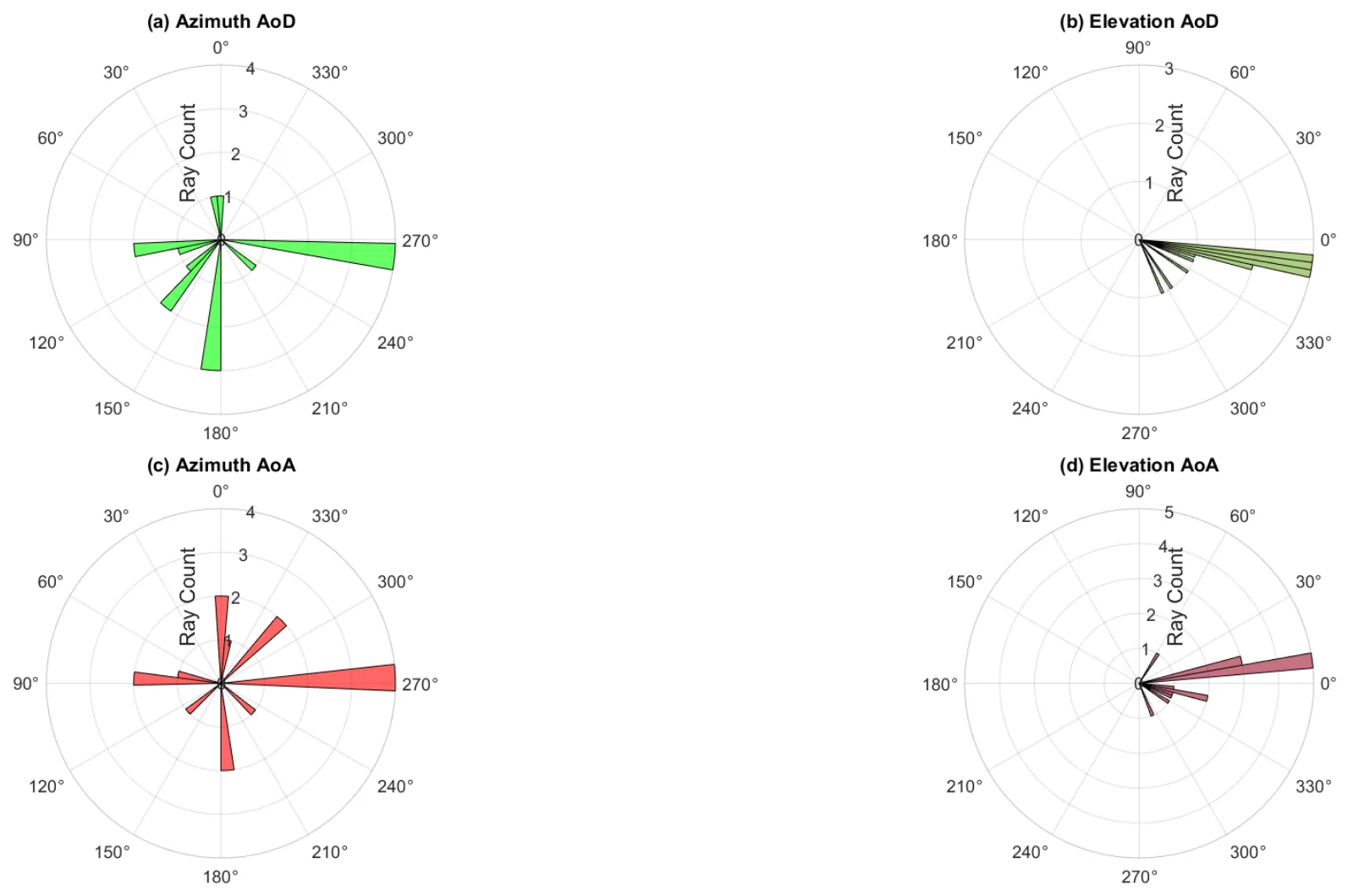
Spatial propagation characteristics of the 140 GHz channel in the smart factory environment, independently evaluating the horizontal and vertical planes. (**a**) Azimuth AoD distribution. (**b**) Elevation AoD distribution. (**c**) Azimuth AoA distribution. (**d**) Elevation AoA distribution.

**Table 1:** Cause-and-effect synthesis of the proposed 140 GHz deterministic propagation framework, mapping mathematical models to physical mechanisms.

Propagation Mechanism & Channel Metric	Associated Mathematical Formulation	Physical Cause in Digital Twin Environment	Deterministic Effect on 6G URLLC Physical-Layer of Performance
Full-Wave Spatial Filtering (Tx)	Complex radiation matrix G(θ,ϕ) and Array Factor integration	The necessity to overcome severe 140 GHz Free Space Path Loss (FSPL) using a highly directive 4×4 array.	Strictly restricts the Angle of Departure ($\sigma_{AoD}$) spread, physically filtering out unnecessary higher-order multi-bounce rays.
Geometry-Based Sub-THz Attenuation	Molecular absorption $e^{-\frac{1}{2}\alpha_{abs}d_{i}}$ and large-scale shadowing $X_{\sigma }$	Atmospheric gas absorption inherent to 140 GHz, combined with heavy NLoS blockages from metallic CNC cells.	Naturally bounds the valid ray subset (L), suppressing long-tail delays below the $P_{sens}$ threshold and increasing spatial sparsity.
Deterministic Multipath Scattering	Coherent superposition $E^{RX}$ via dyadic reflection ${\bar{R}}_{i,r}$ and diffraction ${\bar{D}}_{i,d}$	Specular reflections off perfectly conducting machinery versus penetration/scattering losses through concrete boundaries.	Yields a deterministic Channel Impulse Response (CIR) h(τ,Ω), demonstrating that idealized isotropic transmitter assumptions overestimate scattering.
Spatial-Temporal Dispersion Limit	RMS Delay Spread ($\tau_{RMS}$) and Angular Spread ($\sigma_{\theta }$) models	The constructive/destructive superposition of surviving multipath components (MPCs) arriving at distributed AGVs.	Quantifies temporal dispersion to assess Inter-Symbol Interference (ISI) boundaries, confirming that the constrained delay spread supports physical-layer low-latency requirements.
Bandwidth-Constrained Link Quality	Shannon-Hartley capacity *C* governed by wideband $P_{RX}$ and AWGN $N_{floor}$	10 dBm Tx power restricted by thermal noise boundaries and extreme industrial multipath fading.	Defines the absolute throughput limits for AGV control loops, ensuring ultra-reliable connectivity in dense industrial networks.

**Table 2:** Physical dimensions of the 140 GHz microstrip patch antenna.

Parameter	Symbol	Value (mm)
Substrate Material	Rogers RO3003	-
Dielectric Constant	$\epsilon_{r}$	3.00
Substrate Thickness	*h*	0.127
Patch Width	*W*	0.72
Patch Length	*L*	0.62
Feed Line Width	$W_{f}$	0.316
Inset Feed Depth	$y_{in}$	0.20
Inset Feed Gap	*g*	0.15
Substrate Width/Length	$W_{sub},L_{sub}$	4

**Table 3:** Simulation parameters.

Parameter	Value
Carrier Frequency	140 GHz
UE Antenna Type	Isotropic (0 dBi)
Tx Antenna Type	4×4 Directional Microstrip Patch Array
Transmitter Power	10 dBm
UEs Height	1.0 m above ground level
Tx Height	5.5 m above ground level
Receiver Sensitivity	−90 dBm
Computational Pruning Threshold $\left(P_{prune}\right)$	−120 dBm
System Bandwidth (*B*)	2 GHz
Receiver Noise Figure (NF)	10 dB
Ambient Temperature (*T*)	290 K
Thermal Noise Density	−174 dBm/Hz
Coverage Area	$20\times 20\times 6\;m^{3}$
Material Scatterers	Metallic CNCs, Storage Racks, Concrete Walls
Scenario	Industrial Smart Factory (Indoor Factory-InF)

**Table 4:** Dielectric constant and conductivity of different materials in the digital twin of the industry.

Material	Dielectric Constant ($\epsilon_{r}$)	Conductivity (*σ*) [S/m]	References
Concrete	5.0–6.5	0.40–1.50	ITU-R P.2040-4
Metal	1.0	$10^{7}$	ITU-R P.2040-4
Glass	5.5–7.0	0.10–0.50	ITU-R P.2040-4
Wood	1.5–3.0	0.01–0.10	ITU-R P.2040-4

**Table 5:** Comparative analysis of the 140 GHz sub-THz channel parameters.

Study	Freq. (GHz)	Propagation Condition	Path Loss (dB)	Rx Power (dBm)	Outage (Nb.)	Delay Spread (ns)	AoD RMS (Az./El.) (°)	AoA RMS (Az./El.) (°)
Proposed	140	LoS	90.5	−48.2	0	9.8–11.2	∼2.0/∼0.5	∼15.2/∼2.5
Proposed	140	Moderate NLoS	96.8	−56.4	0	11.5–14.8	∼4.2/∼1.0	∼42.5/∼5.0
Proposed	140	Deep NLoS	106.5	−64.7	0	15.0–21.5	∼6.5°/∼1.5	∼84.1/∼8.0
[9]	142	LoS	–	–	–	7.4	2.3/–	3.7/–
[9]	142	NLoS	–	–	–	9.7	5.1/–	6.3/–

Note: Angular spreads are computed using power-weighted circular statistics to prevent phase wrapping errors. Values are reported independently for Azimuth and Elevation planes (Az./El.). The reported Path Loss represents the omnidirectional spatial channel loss, whereas the $R_{x}$ power is the directional power computed via the AEP-based synthesized 3D radiation pattern of the transmitter array.

**Table 6:** Controlled ablation study evaluating the physical impact of spatial filtering under normalized EIRP conditions (Deep NLoS).

Tx Antenna Configuration	Tx Power (dBm)	Peak Gain (dBi)	Normalized EIRP (dBm)	Max. RMS Delay Spread (ns)	Max. AoA RMS (°)	Channel State/ISI
Theoretical Isotropic	26.86	0.0	26.86	>120.0	∼102.4	Severe Temporal Dispersion
Proposed 4×4 Array	10.0	16.86	26.86	21.5	82.9	Mitigated ISI (Targeted Sector)

**Table 7:** Comparative analysis of the proposed 140 GHz deterministic channel characterization framework against existing propagation studies.

Ref.	Freq. (GHz)	Environment	Antenna Configuration	Modeling Method	RMS Delay Spread (ns)	Outage/ Coverage
[43]	3.5/5.8	Factory Floor	Isotropic/Omni.	Empirical Meas.	>150.0	Unmitigated multipath
[44]	3.7/28	Industrial IoT	Dipole Antenna	Empirical Meas.	120.0–180.0	Broad area coverage
[45]	28	Smart Factory	Standard Horn	Empirical Meas.	40.0–60.0	Sectoral coverage
[46]	60	Indoor Factory	Omnidirectional	Meas. + RT	65.0–85.0	High NLoS susceptibility
[47]	140	Office/Indoor	Directional Horn	Empirical Meas.	15.0–30.0	LoS-dominant link
[48]	300	Data Center	Standard Horn	Empirical Meas.	10.0–25.0	Strict LoS dependence
[9]	142	Factory (InF)	Isotropic (Theo.)	Stochastic Mod.	7.4–9.7	Omnidirectional blockage sensitivity
[49]	28/73 142	Indoor/Outdoor Factory	Directional Horn	Meas. + RT	7.9–29.8	Directional spatial filtering
[50]	140	Indoor Setting	Single Patch	Empirical Meas.	50.0–70.0	High susceptibility to blockage
[23]	140	Industrial Twin	Theoretical Iso.	Deterministic RT	>120.0	Severe temporal dispersion
This work	140	Smart Factory	Custom 4×4 Array	Deterministic SBR	9.8–21.5	Targeted sectoral coverage

## Data Availability

The datasets generated and/or analyzed during this study, including the antenna-array simulation results and factory-environment channel modeling data, are available from the corresponding author upon reasonable request.

## Abbreviations

The following abbreviations are used in this manuscript:

3D	Three-Dimensional
6G	Sixth Generation
AF	Array Factor
AGV	Autonomous Guided Vehicle
AoA	Angle of Arrival
AoD	Angle of Departure
CDF	Cumulative Distribution Function
CNC	Computer Numerical Control
CST	Computer Simulation Technology
dB	Decibel
dBm	Decibel-milliwatt
FSPL	Free Space Path Loss
GHz	Gigahertz
GO	Geometrical Optics
IIoT	Industrial Internet of Things
IoT	Internet of Things
ISI	Inter-Symbol Interference
ITU-R	International Telecommunication Union - Radiocommunication Sector
LoS	Line-of-Sight
MIMO	Multiple-Input Multiple-Output
mmWave	Millimeter Wave
MPC	Multipath Component
NLoS	Non-Line-of-Sight
PDP	Power Delay Profile
RMS	Root Mean Square
Rx	Receiver
SBR	Shooting and Bouncing Rays
SNR	Signal-to-Noise Ratio
Sub-THz	Sub-Terahertz
Tbps	Terabit-per-second
Tx	Transmitter
URLLC	Ultra-Reliable Low-Latency Communications
UTD	Uniform Theory of Diffraction
